# Biomimetic electrospun nanofibrous composites for bone tissue engineering: a review of materials, fabrication, and applications

**DOI:** 10.1186/s12938-026-01596-5

**Published:** 2026-07-23

**Authors:** N. Valarmathi, S. Sumathi, Helen P. Kavitha, M. Swaminathan, Manikandan Krishnamurthy, Manikandan Ayyar, Abdullatif Hakami, Abinet Gosaye Ayanie

**Affiliations:** 1 Department of Chemistry, Vellore Institute of Technology, Vellore, Tamil Nadu 632014 India; 2 Department of Chemistry, Faculty of Engineering and Technology, SRM Institute Science and Technology, Bharathi Salai, Ramapuram, Chennai, Tamil Nadu 600089 India; 3 Nanomaterials Laboratory, Department of Chemistry, Kalasalingam Academy of Research and Education, Krishnankoil, Tamil Nadu 626126 India; 4 Department of Chemistry, Karpagam Academy of Higher Education, Coimbatore, Tamil Nadu 641021 India; 5 Centre for Advanced Solar Cell Technologies, Karpagam Academy of Higher Education, Coimbatore, Tamil Nadu 641021 India; 6 Department of Electrical Engineering, College of Engineering, University of Bisha, P.O. Box 551, 61922 Bisha, Saudi Arabia; 7 Department of Mechanical Engineering, Adama Science and Technology University, 2552 Adama, Ethiopia

**Keywords:** Electrospun nanofibrous, Composites, Bone tissue engineering, Polymer

## Abstract

A flexible and promising method for creating polymeric nanofibrous composites for BTE (bone tissue engineering) is electrospinning**.** The optimal composites should resemble bone’s natural ECM (extracellular matrix), possess suitable surface chemistry, exhibit strong mechanical strength, and demonstrate bioactivity and biocompatibility. Selecting the appropriate material for composite fabrication is a critical initial step in developing a BTE (tissue engineered) product. BTE is a multidisciplinary area that applies engineering concepts to bone-related biological processes. Key considerations in this field include composites, cells, growth factors, and their interfaces in the microenvironment. This review article examines the recent developments in BTE using biomimetic electrospun polymeric biomaterials. It provides an overview of Natural polymer, synthetic polymer, bone structure, biocomposite discusses the ideal characteristics of bone scaffolds, and offers a basic introduction to BTE. The fabrication of bone composites is explored in detail, including various materials and techniques used. Additionally covered the characteristics, benefits, and uses of electrospinning in BTE. The application of electrospun nanofibrous composites composed of natural and synthetic polymers for BTE is thoroughly reviewed and discussed, as well as the biomineralization processes involved. Finally, the study concludes with insights into biomimetic composites for BTE based on electrospun nanofibers.

**Graphical Abstract:**

**Figure:**
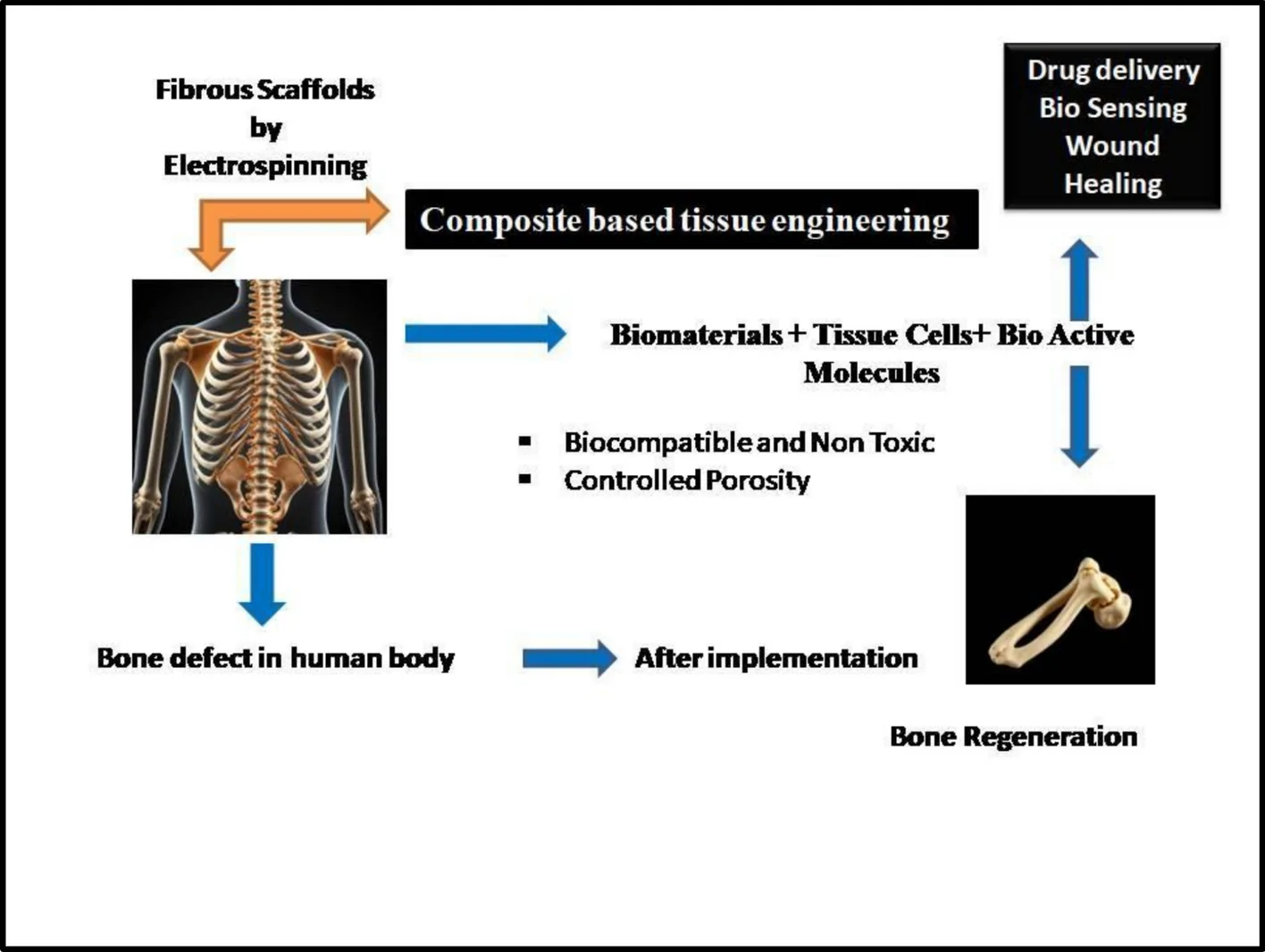

## Highlights

- Electrospun polymeric with bioceramic biomaterials usages were highlighted.
- The electrospinning process, and its applications for bone tissue engineering are also discussed.
- A detailed discussions are presented on the use of electrospun nanofibrous composites made from natural and synthetic polymers for bone tissue engineering applications.

## Introduction

Bone is a rigid, vascularized connective tissue with a complex hierarchical structure that possesses the remarkable ability to regenerate throughout an individual’s life [1]. It ranges in size from macro to nano. It is essential for blood production, motility, support, protection, covering of numerous progenitor cells, homeostasis, and the storage of Ca^2^⁺ and P ions. In addition, it controls the blood’s electrolyte balance [2]. The most common issues that impact both the quantity and quality of human life include aging, accidents, malignancies, infections, and bone abnormalities. Research efforts in understanding cell–matrix interactions which are essential for tissue development have increased as a result of the failure and delay of self-repair for large injuries. Natural grafts have a variety of issues, such as a lack of donor sites, the potential for immune responses, infections, the need for multiple treatments, and pain. As a result, a range of regenerative and synthetic bone repair materials have been developed [3]. The key aim of employing artificial bone scaffolds in orthopedic therapy is to decrease surgical procedures and to lower the risk of infection or immunogenicity [4]. The primary goal of tissue engineering is to repair or protect damaged tissues. This interdisciplinary field achieves its goal by fabricating artificial cellular scaffolds using knowledge derived from cell biology, materials engineering, and biochemistry [5, 6]. Biocomposites are a type of support material that resembles the ECM (extracellular matrix) and provides the platform for cell proliferation, attachment, and differentiation. Biocomposites must be porous, mechanically robust, and biocompatible in order to enhance healing time [5, 7].

The bone ECM is mostly made up of collagen as well as non-collagenous materials, such as proteins and inorganic components, such as nano-hydroxyapatite [8, 9]. Porous biocomposite materials have a significant impact on biological processes, for instance cell migration and cell adhesion, which are involved in the tissue regeneration process in the human body [10, 11]. So far, ceramics and numerous natural and synthetic polymer composites have also been widely employed for BTE. To date, HAP is frequently utilized in bone composite materials and has been found to be highly biomimetic and osteoconductive in nature. However, it has some drawbacks, such as poor mechanical properties. Nowadays, collagen, silk, chitosan, and cellulose and its derivatives are commonly employed in bone tissue engineering (BTE) applications. Moreover, a flexible and promising method for creating polymeric nanofibrous composites for BTE (bone tissue engineering) is electrospinning. The optimal composites should resemble bone’s natural ECM (extracellular matrix), possess suitable surface chemistry, exhibit strong mechanical strength, and demonstrate bioactivity and biocompatibility. Selecting the appropriate material for composite fabrication is a critical initial step in developing a BTE (tissue engineered) product. Consequently, electrospun nanofibers are being extensively researched for use in diverse areas, such as drug delivery systems, tissue engineering, biosensors, wound dressings, filtration membranes, and enzyme immobilization [12, 13].

A strong electric field is utilized to generate micro-to-nano-sized fibers from a polymer solution, achieving variable porosity. In contrast to conventional techniques, electrospun nonwoven nanofibrous mats are able to accurately mimic the constituent parts of the extracellular matrix [14]. With a focus on BTE, Khorshidi et al. [15] examined the most current recommendations for employing the electrospinning technique to make nanofibrous scaffolds. Recent studies have looked at the production, composition, and application of electrospun polymer nanocomposites in osteochondral tissue engineering (TE). The spinnability of collagen-like biomimetic composites for BTE applications was examined by Bazrafshan and Stylios [16]. Soares et al. [17] illustrated the simulated utilization of electrospinning and electrospraying methods to synthesize bio-based polymer electrospun nanofibrous materials. Ding et al. [18] recently published a review on electrospun polymer biomaterials used for wound healing, sensors, reinforcement, and other applications.

Despite the growing number of publications on electrospun scaffolds for BTE [15–18], the current literature suffers from three unaddressed gaps. First, most studies report “successful biomineralization” without distinguishing between surface-bound mineral (which is mechanically weak and often detaches) vs. intrafibrillar mineral (which truly mimics natural bone). Second, electrospun mats typically have pore sizes < 20 µm, preventing meaningful cell infiltration beyond 50–100 µm, yet they are routinely described as “3D scaffolds”. Third, mechanical property claims rarely benchmark against actual human bone values (trabecular: 100–2000 MPa compressive modulus), leading to overstatements of clinical relevance. Existing review articles have cataloged materials and methods but have not systematically analyzed these specific failures or proposed quantitative benchmarks for success.

This review uniquely addresses these gaps in three ways: (1) it applies a consistent mineral localization framework to distinguish surface vs. intrafibrillar mineralization. (2) It critically evaluates reported cell infiltration depths and identifies which fabrication strategies actually achieve true 3D colonization. (3) It normalizes all mechanical data to human trabecular bone standards, explicitly identifying which scaffold compositions fall short and which approach clinical relevance. By doing so, this review moves beyond descriptive cataloging to provide actionable design guidelines for the next generation of biomimetic electrospun BTE scaffolds. Critically, this review moves beyond descriptive cataloging to provide: (1) a quantitative comparison of mechanical–biological performance trade-offs across natural polymers, synthetic polymers, and their HAP composites; (2) identification of contradictions in the literature (e.g., conflicting reports on optimal fiber diameter for osteogenesis); and (3) a framework for designing hierarchical scaffolds that decouple porosity from mechanical failure. By explicitly mapping, where current electrospun biocomposites fail to meet the dual demands of large bone defects, this review aims to guide the rational design of next-generation scaffolds that balance biomimetic bioactivity with load-bearing mechanical robustness—a prerequisite for clinical translation beyond small animal models.

In order to aid BTE, this review will present the most current developments in electrospun polymeric scaffolds. The initial portion of this review covers tissue engineering, BTE, bone structure, and the requirements for acceptable bone composites. The characteristics of several identified biomaterials and composite production procedures are then explored. The advantages, benefits, and applications of the electrospinning method for BTE are discussed. Finally, we have concluded our investigation into the potential applications and challenges of electrospun nanofiber-based biomimetic composites for BTE applications. Before examining specific electrospun composites and their performance, it is essential to establish the foundational principles of tissue engineering and the unique requirements of bone as a target tissue. The following sections provide an overview of tissue engineering strategies, the structure and composition of human bone, and the critical design criteria that biomimetic scaffolds must satisfy. This foundational understanding informs the subsequent discussion of biomaterials, fabrication techniques, and the quantitative comparisons that form the core of this review.

## Tissue engineering (TE)

**Fig. 1:**
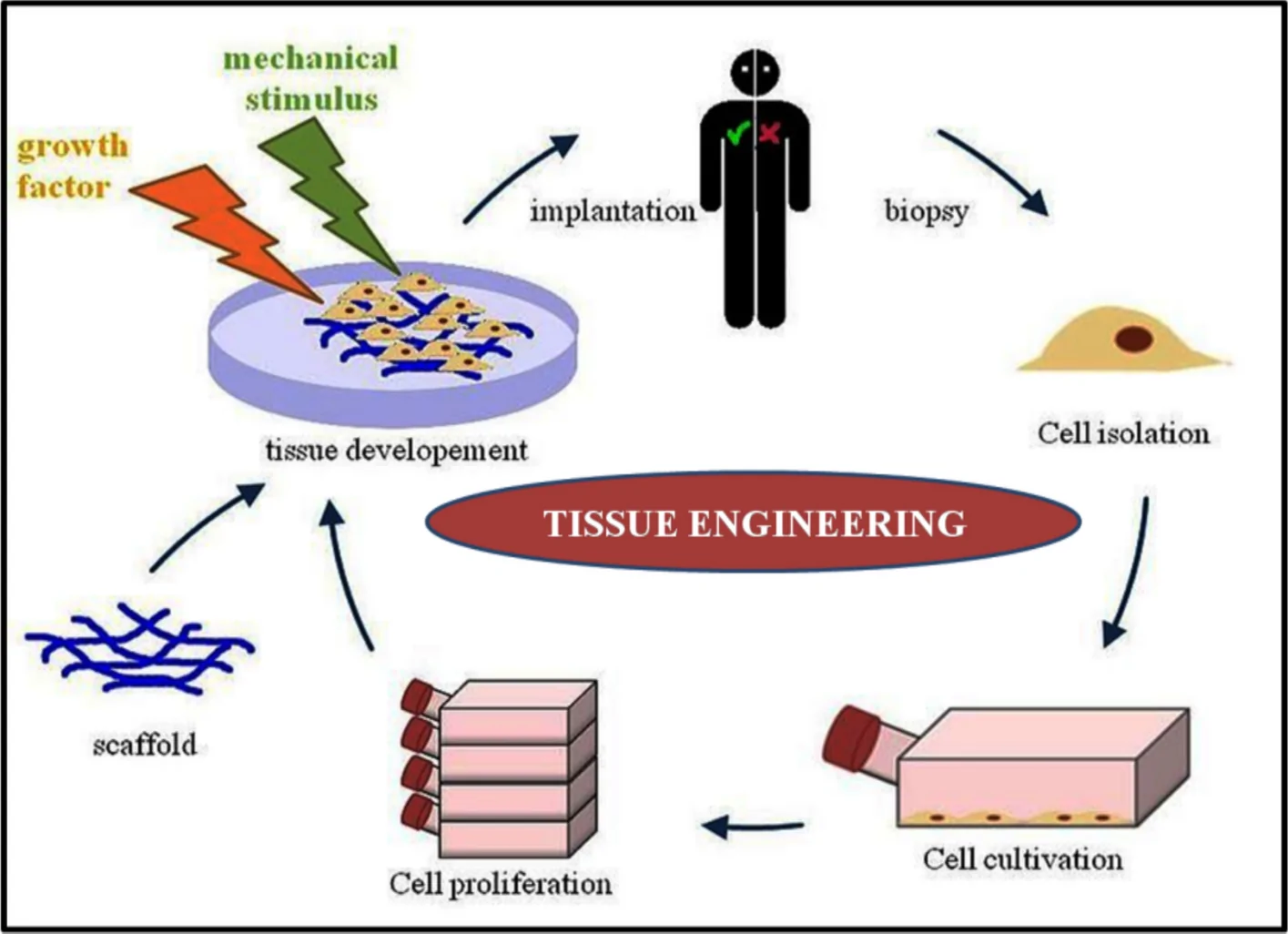
Concepts of tissue engineering in biomedical applications [22, 23]

Tissue engineering (TE) uses concepts from biology and engineering to develop biological substitutes that improve, preserve, or repair tissue function. Several methods are utilized to create new tissue, among them: (i) isolation of cells to replace cells that provide essential functions [19]; (ii) distributing compounds that promote the formation of new tissue, such as growth factors, to specific areas [19, 20]; and (iii) growing cells in 3D biocomposites [19, 21]. The first and second methods have certain limitations, since they are only appropriate for minor abnormalities and there is a risk that transplanted cells will be rejected due to immune rejection. As a result, the third technique has been more widely adopted in research, particularly in the field of BTE. Moreover, scaffolds must possess surface properties, such as those that enable cell adhesion, growth, and proliferation [22], as shown in Fig. 1.

Figure 1 shows that the tissue engineering serves as the central hub. It directly leads to tissue development as the final outcome. To achieve this, the figure links several inputs and processes: mechanical stimulus, growth factor, implantation, biopsy, and scaffold are all connected to the core. In addition, cell proliferation and cell cultivation represent key cellular activities that likely occur within or alongside the scaffold and growth factors. While the principles of tissue engineering apply broadly to various organs and tissues, bone presents unique challenges and opportunities due to its complex hierarchical structure, vascularization, and load-bearing requirements. The following section focuses specifically on bone tissue engineering (BTE), discussing its clinical relevance, and the specific demands that bone defects place on scaffold design.

### Bone tissue engineering (BTE)

**Fig. 2:**
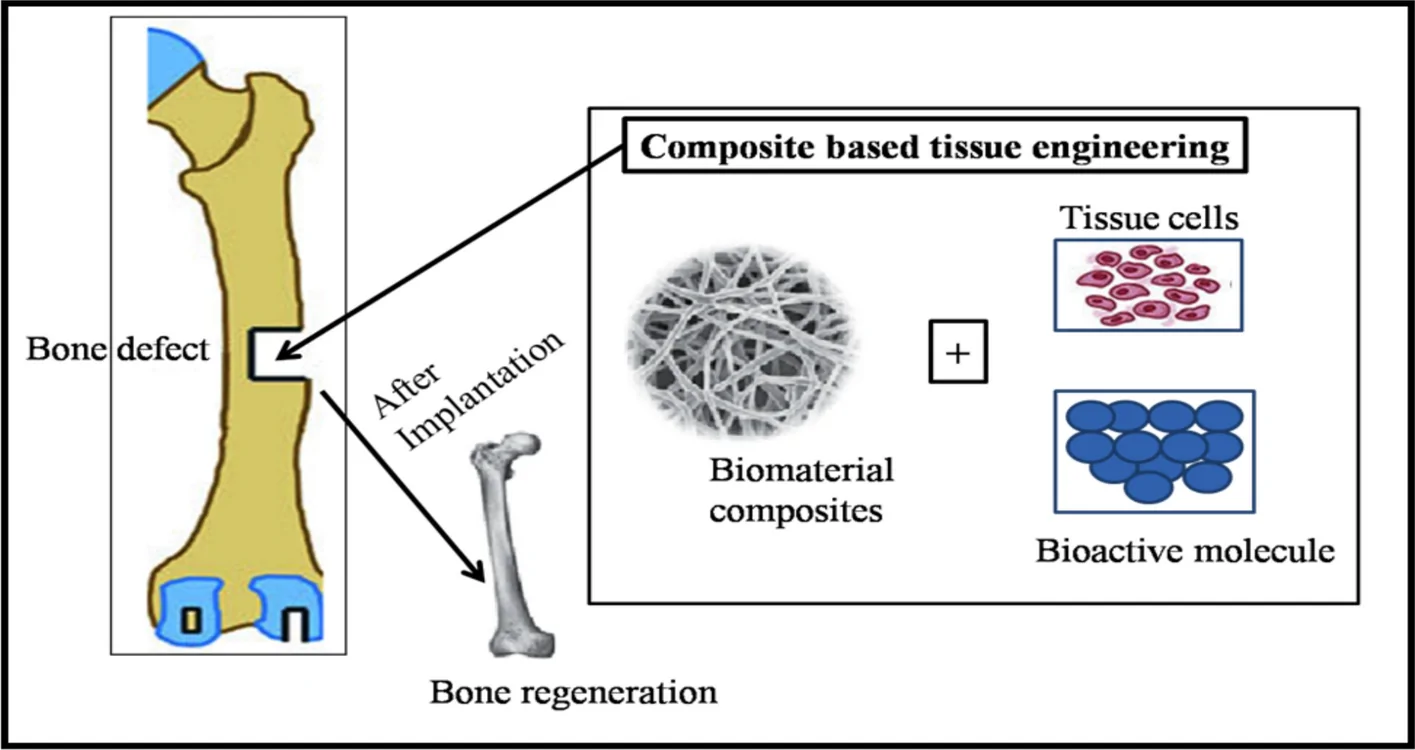
Schematic representation of the core components and process of bone tissue engineering (BTE)

BTE is found to be a very good method for bone structure improvement, bone strength enhancement, and development of tissue functions (see Fig. 2). Bone is the next most commonly transplanted tissue worldwide. The prevalence of bone disorders and diseases has increased everywhere in the world. These days, bone repair is required for bone problems, including infections, tumors, and trauma [24]. Although bone has the ability to heal itself, there are situations, where complete bone repair is not possible and needs to be stimulated [25]. Many individuals with bone irregularities require bone grafts or replacements. As a result, the field of bone regeneration within tissue engineering has become an attractive area. The objective of the BTE domain is to optimize biological resources that will increase new bone regeneration [26]. In the past few years, BTE has become one of the most challenging tasks for treating bone injuries in the human body. In recent times, biomaterials in the form of whole prostheses, hydrogels, and scaffolds have been used to treat bone injuries caused by osteoporosis, osteoarthritis, neoplasms, and fractures [10, 27]. BTE has achieved a number of successes in animals when using biomaterials and cells selected from primary mature osteoblasts and bone marrow mesenchymal stem cells. In fact, mesenchymal stem cells have been shown to have potential for bone repair in research since the 1980s.

Figure 2 presents a targeted application of tissue engineering specifically for bone defect repair. The central concept is composite-based tissue engineering, which implies that no single material is sufficient—rather, a combination of biomaterials (e.g., polymers, ceramics, or metals) is required to mimic native bone’s complex structure and function. Three key components work synergistically: tissue cells (osteoblasts or stem cells), Biomaterial composites (structural scaffolding), and Bioactive molecules (growth factors, cytokines, or drugs). Their interplay is crucial—cells need a biocompatible scaffold to adhere and proliferate, while bioactive molecules direct differentiation and matrix production. The temporal sequence is implicit: starting from a bone defect (pathological state), the composite construct is prepared, followed by After Implantation into the host. This stage is critical, as it introduces in vivo challenges, such as immune response, vascularization, and mechanical loading. The ultimate goal is bone regeneration—not just filling the defect but restoring native architecture and function.

Although experimental approaches are now being investigated, orthopedic surgeons still rely on conventional methods of replacement; however, some of the newer techniques are gradually receiving approval from medical professionals. The predictable treatments for bone defects involve bone grafting by utilizing autografts (from the patient’s own bone, such as from the hip) or allografts (from a donor of the same species) to fill bone deficiencies through surgery. Hip fracture rates rose by roughly 500% in Singapore and by around 300% in Hong Kong during the previous four decades. In Japan, there are already 12 million cases of osteoporosis due to an aging population (over 75 years), which will provide a large market for bone implantation in the future [28].

In 2010, the US market had spent $1.3 billion on alternatives to bone transplants, and by 2017 it is predicted to reach $2.2 billion [12]. In the European market, bone implant sales were over $177.1 million in 2010, and by 2017, they are projected to rise by up to 17.3%. Understanding the hierarchical structure and chemical composition of natural bone—from collagen fibrils to HAP crystals provides the design criteria for synthetic biomaterials, which are discussed next.

### Structure of human bone

**Fig. 3:**
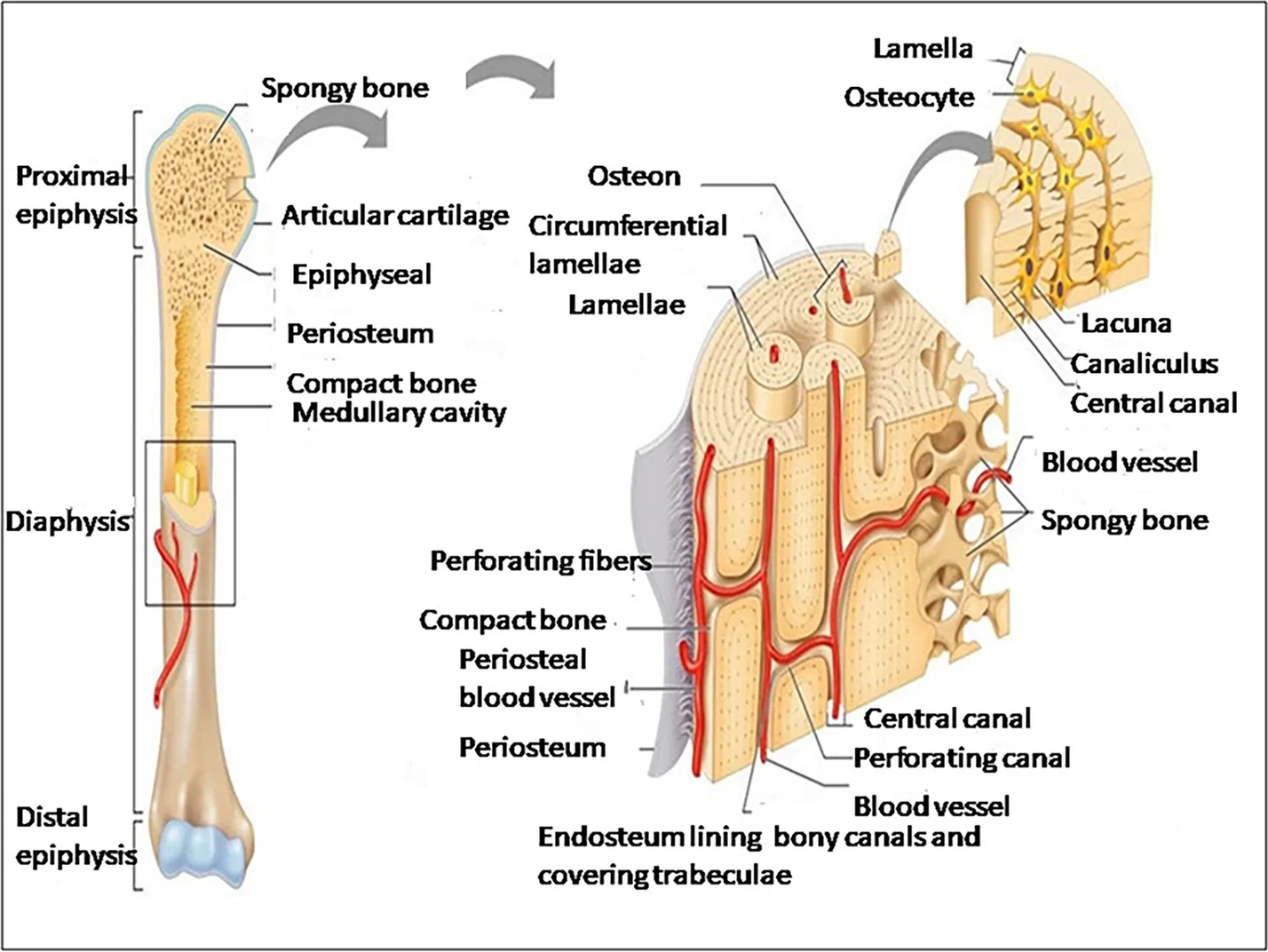
Long bone cross section [31]

The most important requirement in bone structure is it must the mimic the natural bone’s ECM macro–nano-structure, and its materials compositions. The most important thing to note is that natural bones have a macro-to-nano-structure with good biomechanical properties. They have a complex array of hierarchical structures starting from the nanometer scale and continuously growing to the macroscopic level [29, 30]. So far, two types of bone formations have been identified: (i) cancellous or spongy bone and (ii) compact or cortical bone. Compact bone is found in the shafts of long bones and consists of a variety of cylindrical units [31], as shown in Fig. 3. It is important to note that it contains Haversian canals, which are mainly enclosed by concentric lamellae tissue.

Figure 3 illustrates the anatomy of a long bone alongside its microscopic structure and vascular supply. The left diagram labels the gross features, including the proximal epiphysis, distal epiphysis, diaphysis, spongy bone, articular cartilage, epiphyseal line, periosteum, compact bone, and medullary cavity. The right diagram focuses on the microscopic organization, showing an osteon (Haversian system) with components, such as osteocyte, circumferential lamella, lamella, lacuna, canaliculus, and the central (Haversian) canal, along with a blood vessel continuing into the medullary cavity containing marrow and spongy bone. The middle diagram details the vascular network, including periosteal blood vessel, periosteum, perforating (Volkmann’s) canal, blood vessel, and endosteum lining bony canals and covering trabeculae.

With the aid of intercommunicating systems, the Haversian canals are primarily used to transport metabolic substances through small cavities. When viewed at a microscopic level, cancellous bone is mostly made up of a cellular structure and is present in both long and flat bones. It is mostly composed of a complex of interconnecting trabeculae that aid in creating spaces for communication [12]. In addition, trabecular bone [32] lacks Haversian canals or hollows and is essentially closed (Fig. 3).

The image illustrates the structural hierarchy of bone, starting at the molecular level with the collagen molecule. These molecules form a tropocollagen triple helix, which measures approximately 15 nm in one dimension and 50 nm × 25 nm × 3 nm overall. Associated with this is the hydroxyapatite (HA) nanocrystal. At a larger scale, osteons (around 100 μm in size) are shown, along with osteons and Haversian canals.

**Fig. 4:**
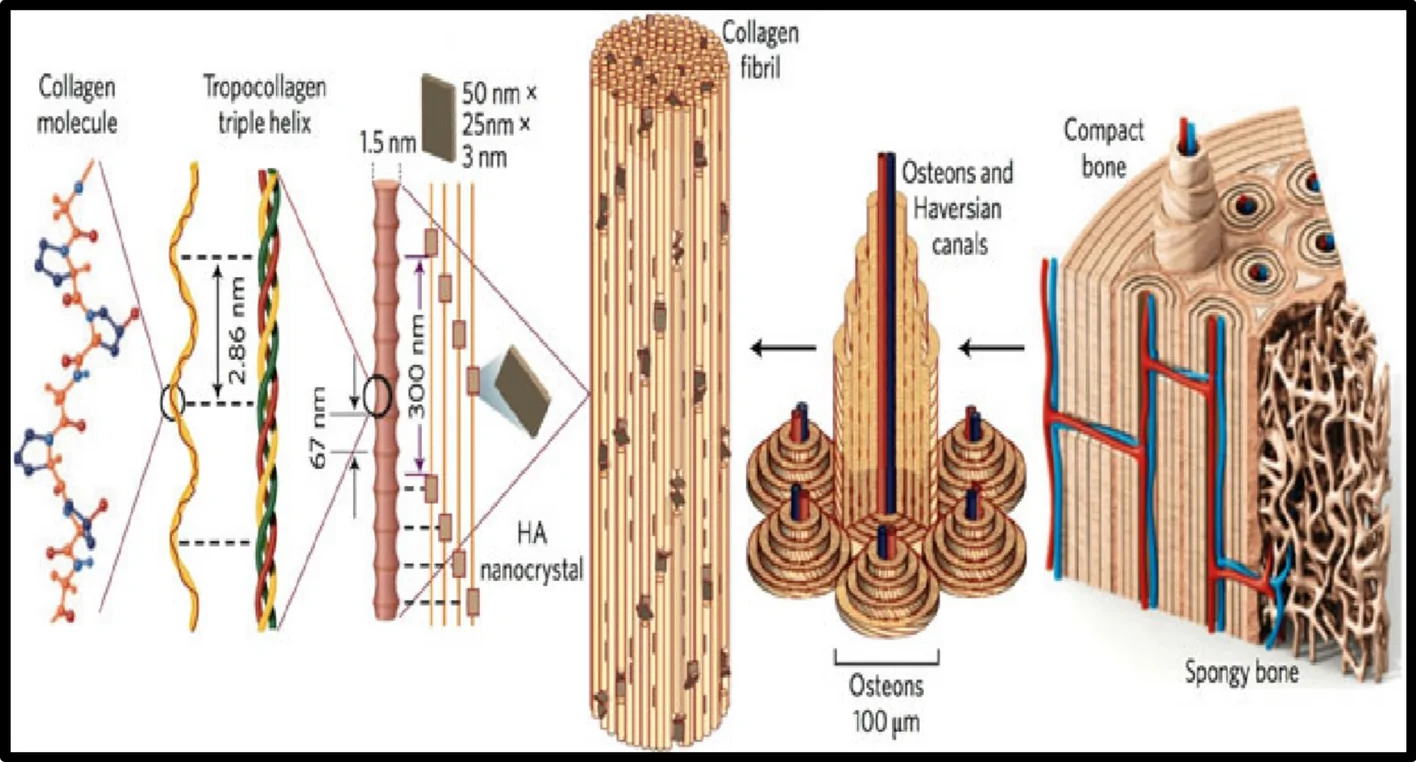
Micro-to-nano-scale bone structure is organized hierarchically [12]

Tropocollagen molecules are made up of collagen fibrils ranging in size from 100 to 2000 nm. The configuration of collagen fibrils aids in the microscale formation of lamellae [33] and their mineralization. Most of the tropocollagen is made up of hand helices (Fig. 4). The collagen fibrils are the main sites, where nucleation of bone mineral crystals occurs. These crystals are mainly composed of hydroxyapatite (HAP). It has been reported that the dimensions of HAP crystals can range from 1.5 to 5 nm in thickness, 20 nm in width, and 40 to 60 nm in length, depending mainly on the synthesis method [12, 34]. Natural human bones are made of HAP crystals, which provide mechanical strength and density to natural bone. It is essential to mention that bone consists of fluid and solid phases. It is very hard due to the organic extracellular collagen, which is permeated with inorganic minerals, such as Ca/P, generally HAP. Calcium and phosphorus comprise approximately 65–70% of the dry weight of natural bone. Although the ratio of calcium to phosphorus (Ca/P) in normal bone is 1.67, it can vary from 1.37 to 1.87 due to the presence of additional mineral ions (Table 1) [35]. Collagen fibers make up between 25% and 30% of the bone matrix. The fact that bone contains more type-I collagen and comparatively less type-IV collagen is instructive. Type-I collagen molecules come together to form tropocollagen, which is then surrounded by mineralized collagen fibers. Tropocollagen also contains proteins in the form of glycosaminoglycans (GAGs), polysaccharides, and complex macromolecules called proteoglycans. The inorganic component gives bone its hardness, while the organic component gives bone its flexibility [36, 37]. Table 1 shows the Human bone’s chemical composition. Understanding the hierarchical structure and chemical composition of natural bone—from collagen fibrils to HAP crystals—provides the design criteria for synthetic biomaterials, which are discussed next.

**Table 1:** Human bone’s chemical composition [35]

S.No	Elements	wt%
1	Calcium	34.8
2	Phosphorus	15.2
3	Sodium	0.9
4	Magnesium	0.72
5	Potassium	0.03
6	Fluoride	0.03
7	Chloride	0.13
8	Carbonate	7.4
9	Pyrophosphate	0.07
10	Water	10.00

## Biomaterials utilized in BTE (Bone tissue engineering)

**Fig. 5:**
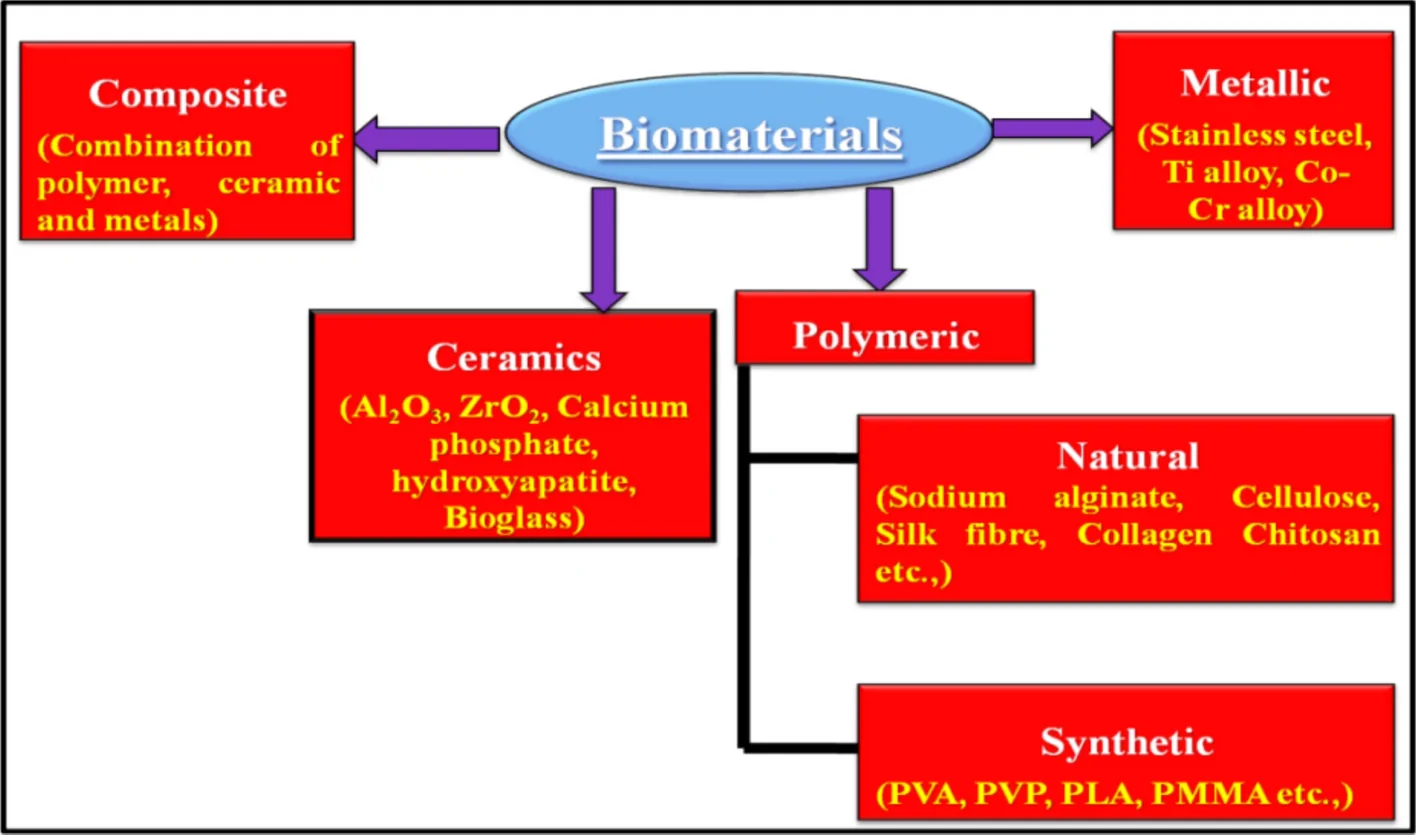
Classification of biomaterials for bone tissue engineering

The final generated, sterilized, and biocompatible form of a material used in bio-devices is referred to as a biomaterial. Williams et al. provide the most well-known definition of biomaterial and biocompatibility: “A biomaterial is a nonviable material used in a medical device that communicates with biological systems”. It should be highlighted that without expertise in this field, any researcher would be unable to produce and synthesize a biomaterial suitable for the “specific use” mentioned in the latter definition. As a result, the term “specialization” needs to be defined further. Each artificial implant has certain performance standards based on the physical properties of the biomaterial. Moreover, “any material that has interacted with biological systems for therapeutic (repair or replace a bodily tissue function) or diagnostic purposes” is called a biomaterial [38]. To date various synthetic biomaterials for BTE applications were developed and investigated. In addition, these biomaterials were classified as polymeric, ceramic, metallic, and composite (Fig. 5). They were categorized based on how they react with or stimulate biological tissue. Physiologically inert, biomimetic, bioactive, and biodegradable substances are all considered biomaterials. Biomaterials are materials that interact with human tissues and biological fluids and are essential for hand surgery involving bones, as well as hip or knee replacement [39].Among these four classes of biomaterials, ceramics—particularly calcium phosphate-based bioceramics—have gained significant attention due to their compositional similarity to the mineral phase of natural bone and their excellent osteoconductive properties, as discussed below.

## Bioceramics

Bioceramic biomaterials offer several distinguishing characteristics, including strong biocompatibility, osteoconductivity, and bioactivity. Ceramics are effective biomaterials for bone grafting due to these qualities. Since the mineral phase of human bone and teeth is mostly composed of calcium and phosphate ions, Ca/P ceramics are widely utilized as bioceramics today. Due to their high solubility and acidity, Ca/P ratios of less than one imply that they are unsuitable as biomaterials. Table 2 shows the five different forms of Ca/P bioceramics used as biomaterials [39]. Despite the clinical utility of β-TCP, its rapid resorption and incomplete bone replacement have led researchers to investigate more stable and osteoconductive calcium phosphate ceramics, with hydroxyapatite emerging as the gold standard due to its chemical and structural similarity to native bone mineral.

**Table 2:** Ratio of Ca/P utilized as biomaterials

S.No	Name and chemical formula	Ca/P ratio
1	ACP–Ca_10-x_H_2x_(PO_4_)_6_(OH)_2_	1.5
2	OCP–Ca_3_H_2_(PO_4_)_6.5_H_2_O	1.33
3	TCP–Ca_3_(PO_4_)_2_	1.50
4	HAP–Ca_10_(PO_4_)_6_(OH)_2_	1.67
5	TetCP–Ca_4_O(PO_4_)_2_	2.0

### Beta-tricalcium phosphate (β-TCP)

One of the first calcium phosphate substances to be utilized as a bone transplant material was beta-tricalcium phosphate (β-TCP). β-TCP is structurally porous and exhibits compressive and tensile strengths comparable to cancellous bone. Beta-tricalcium phosphate is reabsorbed over a period of 6–18 months through dissolution and fragmentation. Nevertheless, β-TCP replenishment by bone is not evenly distributed. When β-TCP is reabsorbed, less bone volume is formed. As a result, β-TCP has been used therapeutically as a complement to other less reabsorbable bone graft substitutes. Despite the clinical utility of β-TCP, its rapid resorption and incomplete bone replacement limit its effectiveness as a standalone graft. Consequently, hydroxyapatite (HAP) has become the most widely studied and applied calcium phosphate ceramic for bone tissue engineering, owing to its superior stability, osteoconductivity, and compositional similarity to natural bone mineral.

### Hydroxyapatite

The mineral phase of natural bone is predominantly composed of hydroxyapatite (Ca_5_(PO_4_)_3_(OH), HAP). Because of this, HAP is commonly used to create synthetic bone composites that replicate the natural mineralogical environment of healthy bone tissue [40]. Out of all the mineral phases present in bone, only HAP and dicalcium phosphate (DCP) are chemically stable at body temperature and pH. Crystallographic and chemical tests show that synthetic HAP is comparable to naturally occurring HAP [41]. The term “ceramic” refers to the method used to sinter hydroxyapatite crystals at temperatures ranging from 700 to 1300 °C to produce a highly crystalline compound. Ceramic HAP formulations resist in vivo reabsorption, which occurs at a rate of 1–2% per year.

Synthetic HAP is available in porous ceramic forms. Ca_4_(PO_4_)_2_O and CaHPO_4_, sold as Bone Source (Leibinger, Dallas, TX, USA), form a thick paste when mixed with water. This sets in 15 min via an isothermic process, and after 4 h, the cement hardens into microporous HAP. The implant must be kept clear of fluid collection throughout this conversion stage, since fluid will disintegrate the implant and result in particulate setting. After being transformed into HAP, it is no longer vulnerable to disintegration. This material has been utilized effectively in sinus repair and cranioplasty surgeries. HAP bioceramics exhibit better bioactivity and higher resorbability. The addition of hydroxyapatite (HAP) to synthetic bone scaffolds is known to improve cellular adhesion, promote osteointegration, and provide an optimal osteoconductive matrix for new bone growth [42].

Hydroxyapatite (HAP), with the chemical formula Ca_5_(PO_4_)_3_(OH), is the most thermodynamically stable calcium phosphate under physiological conditions (pH ~ 7.4). It possesses a hexagonal crystal structure with unit cell dimensions of a = 0.943 nm and c = 0.688 nm and a Ca/P molar ratio of 1.67. This mineral constitutes the inorganic phase of bone, where it forms a strong chemical bond with the organic matrix to create a rigid, crystalline composite tissue [43]. Given the critical role of HAP in bone tissue engineering, various synthesis methods have been developed to produce HAP with tailored properties—including crystallinity, particle size, and morphology—each offering distinct advantages for specific biomedical applications.

#### Methods of HAP preparation

Versatile methods for HAP synthesis are available which could be used for different biomedical applications**.** A number of the preparation methods were given below.

The most common and extensively utilized approach for the preparation of HAP is the precipitation process. This method allows for the generation of a relatively substantial amount of HAP at a reasonable cost without the use of organic solvents. Using this method, the size and form of the ceramic (HAP) particles are largely determined by the reaction temperature and the rate at which orthophosphoric acid is added. The sol–gel technique is an excellent method for producing nanophasic HAP due to its ability to tightly regulate production parameters. This method allows the molecular mixing of calcium and phosphorus, which can considerably enhance the structural properties of HAP. Research conducted in both laboratory settings (in vitro) and living organisms (in vivo) indicates that hydroxyapatite (HAP) synthesized via the sol–gel method enhances stability and improves integration at the interface between bone implants and natural bone tissue. The twentieth century saw the hydrothermal method become a cornerstone technology for the synthesis of advanced materials. Using this technology, numerous ceramic materials, including HAP, can be manufactured. Hydrothermal synthesis is a technique for crystallizing ceramic materials directly from aqueous solutions. This process utilizes elevated temperatures (typically above 125 °C) and pressures (greater than 100 kPa) to facilitate reactions from either single or heterogeneous phases. To promote the spontaneous nucleation of bone-like hydroxyapatite (HAP), a metastable simulated body fluid (SBF) is used. This solution, which mirrors the inorganic salt composition of human blood plasma, facilitates the growth of nanocrystalline, carbonated HAP at standard physiological temperature and pH**.** An electrodeposition process conducted at physiological pH can generate an ultrafine-grained nanophase HAP coating. The electrolyte for this process is dilute, with concentrations of 6.1 × 10^–4^ M for Ca^2+^ ions and 3.6 × 10^–4^ M for PO_4_^3−^ ions, sourced from Ca(NO_3_)_2_ and NH_4_H_2_PO_4_ precursors. To increase the electrolyte’s ionic strength, sodium nitrate (NaNO_3_) is added.

### Metals

As permanent prostheses, metallic implants have been used for hip prostheses and dental implants, as well as for short-term implants such as plates, pins, screws, and rods for bone fracture repair. Common materials employed for medical implants include titanium and its alloys, stainless steel, and cobalt–chromium alloys [44]. Bone in growth and implant integration can be promoted by using bioactive ceramic coatings, porous bead surfaces, or cement for fixation. One disadvantage of using metallic implants is that a second surgery is required to remove temporary implants, as well as the negative tissue reaction caused by ions released from permanently implanted devices. Ceramics and metals, on the other hand, have significant limitations, such as slow biodegradability and weak mechanical characteristics. Because metals and ceramics have continued to be the most popular bone substitutes in recent years, many researchers have been focusing on the development of polymeric biocomposite materials. Despite their widespread use, metals and ceramics face limitations such as slow biodegradability and mismatch with native bone stiffness. Consequently, researchers have increasingly turned to polymer-based composites, which offer tunable degradation and mechanical properties. Despite their widespread use, metals and ceramics face limitations such as slow biodegradability and mismatch with native bone stiffness. Consequently, researchers have increasingly turned to polymer-based composites, which offer tunable degradation and mechanical properties.

### Polymer and polymer composites

Since they can imitate the natural ECM, in the future, polymers (synthetic and natural) are the potential biomaterials used for BTE applications [22]. Polymeric biomaterials are particularly adaptable and may be used to alter the structure, form, and biodegradability of bone tissue [45] because of their chemical and physical flexibility. Here, two types of polymers are discussed, such as (i) biopolymers and (ii) synthetic polymers. Polymers are macromolecules consisting of long, repeating chains of monomers. They are used to build 3D composites in tissue engineering applications. In addition, polymers utilized to produce biomaterials are classified into three types: natural, biodegradable synthetic, and non-biodegradable synthetic. Proteins, polysaccharides, and polynucleotides are examples of natural polymers that may be broken down enzymatically or hydrolytically. Natural polymers are essential components of both plant and animal biological structures. These macromolecules include keratin, which is a key structural protein in hair; chitin, which forms the exoskeletons of crabs and other arthropods; silk, which is produced by spiders for their webs; collagen, a fundamental protein in connective tissues; and cellulose, the primary structural polymer in plant cell walls [46]. Due to their outstanding biocompatibility and favorable mechanical properties, biomaterials such as fibrin, collagen, hyaluronic acid, chitosan, agarose, silk proteins, and alginate are commonly utilized in engineering bone and cartilage tissues [47].

Natural polymers promote cell adhesion and proliferation through their built-in templates. While beneficial for tissue growth, these materials can also stimulate an immune reaction. A key characteristic is their highly organized structure, which includes specific ligands for cell signaling—extracellular molecules that attach to cell receptors with ease. The bulk of the ECM is composed of natural polymers that are biocompatible, naturally remodeling, and capable of promoting cell adhesion. A general overview of the polymeric substances mentioned above is provided to justify their utility in biomedical applications, particularly BTE. Synthetic polymers offer several benefits, including tunable porosity, customizable mechanical strength, controllable degradation rates, and variable topographies, all of which can be precisely engineered during the synthesis process. Under regulated conditions, synthetic polymers may be created in large quantities in a predictable and repeatable manner. Nevertheless, one of their significant limitations is that they lack cell recognition areas for osteoconductivity and adhesion. These constraints can be overcome by employing additional bioactive complementary techniques and cell-binding motifs, such as arginylglycylaspartic acid (RGD). Among natural polymers, collagen stands out as the most abundant structural protein in the human body and a primary component of the bone extracellular matrix, making it an ideal candidate for bone tissue engineering scaffolds, as discussed below.

### Collagen

**Fig. 6:**
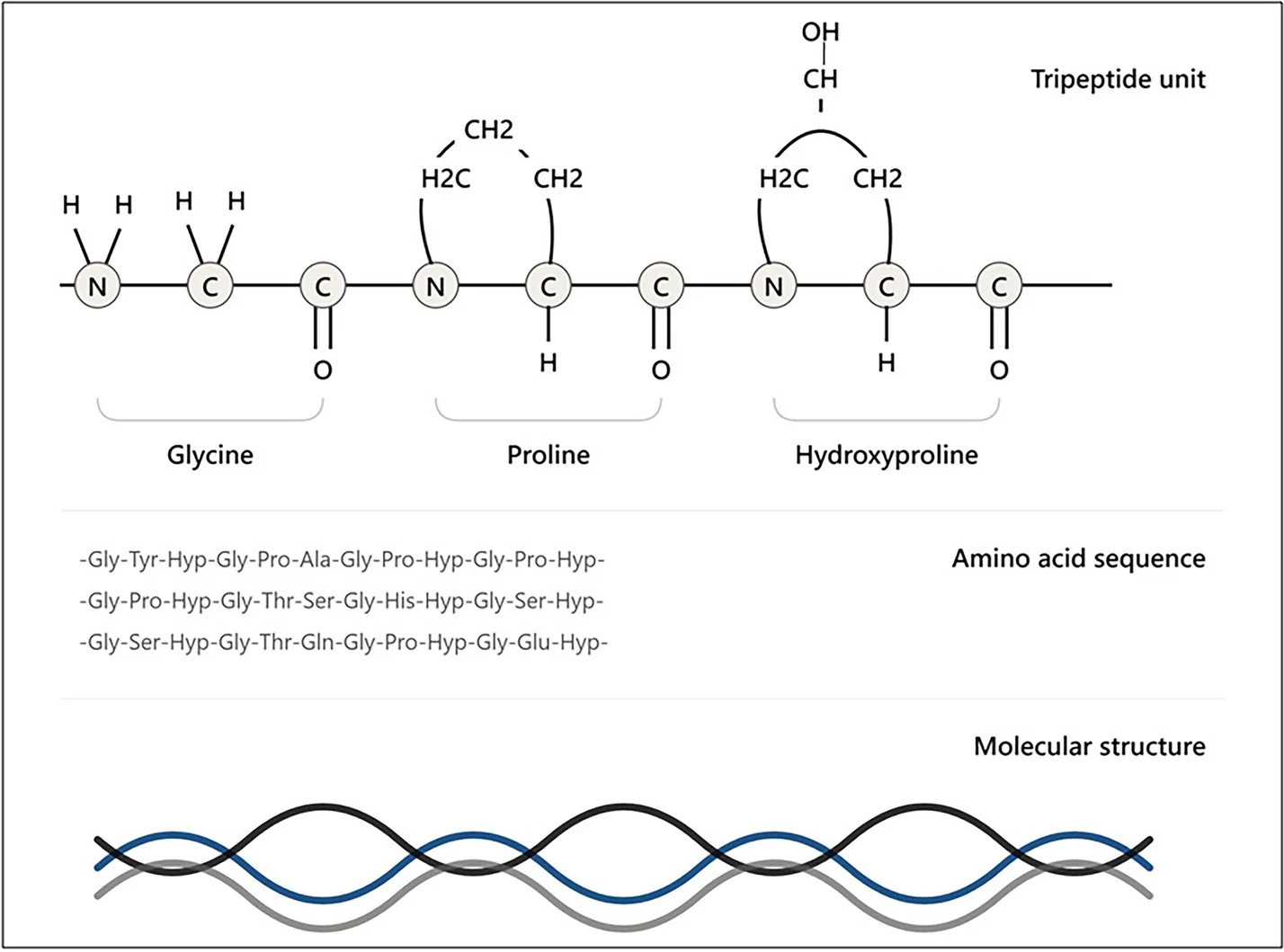
Collagen’s chemical structure

Collagen is the most prevalent fibrous protein in the body and the most significant component of the ECM of all creatures, including sponges and invertebrates. Collagen fibrils provide mechanical properties to every connective tissue [48]. Figure 6 depicts collagen’s chemical structure. As a natural and abundant component of the extracellular matrix (ECM) in human tissues, including bone, skin, and ligaments, collagen is highly biocompatible, biodegradable, non-cytotoxic, and non-immunogenic. These properties make it exceptionally valuable for a wide range of biomedical applications. Therefore, collagen is frequently utilized with ceramics such as HAP for applications involving bone tissue engineering [49].

According to recent studies, nanofibrous scaffolds combining collagen, hydroxyapatite (HAP), and chitosan have been shown to effectively promote the differentiation of mesenchymal stem cells into osteoblasts [50]. Jing et al. [51] presented the preparation of PCL/collagen biocomposite nanofibers used for tissue engineering applications. Kazanci [52] presented a 3D electrospun collagen composite prepared by simply folding a nanofibrous mat. A comparative analysis of collagen nanofibers made with various solvents and stabilizing agents was presented by Fiorani et al. [53]. While collagen is widely used for bone tissue engineering, other natural polymers such as chitosan offer complementary advantages, including inherent antibacterial activity and positive surface charge, which enable unique interactions with negatively charged extracellular matrix components.

#### Chitosan

**Fig. 7:**
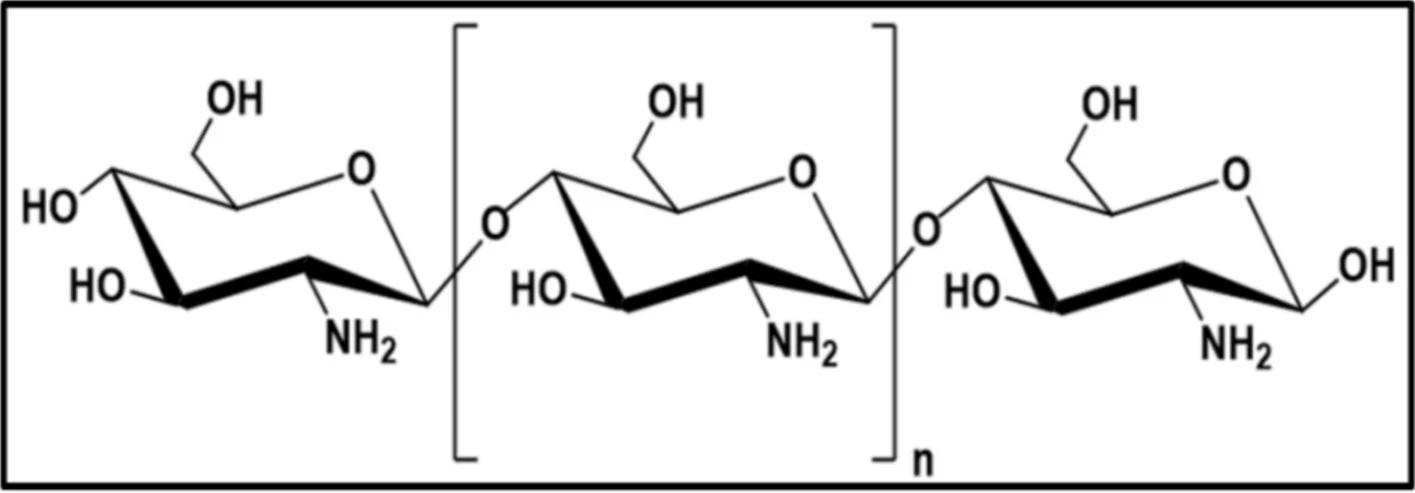
Chemical structure of chitosan [54]

The linear polysaccharide chitosan consists of a random arrangement of two monomeric units: N-acetyl-D-glucosamine and D-glucosamine, which are connected via β-(1 → 4) glycosidic linkages (see Fig. 7) [54]. Chitosan is desirable as a component of bone composites, because it promotes osteoblast cell adhesion and proliferation, in addition to the development of mineralized bone matrix [55]. This biopolymer is highly valuable in biomedicine due to its excellent biocompatibility—including low toxicity and non-immunogenicity—coupled with its biodegradability, inherent antibacterial activity, and capacity for cell ingrowth. Chitosan is the only positively charged biopolymer that can interact with negatively charged structural molecules and polymers in the ECM. Furthermore, chitosan can be combined with other polymers and inorganic materials to enhance the functional properties of composites used in bone defect repair. Another widely studied natural polymer derived from collagen is gelatin, which retains collagen’s bioactivity while offering improved solubility and processability for electrospinning, though it requires specific solvent systems to form stable nanofibers.

#### Gelatin

Gelatin is made from a combination of proteins and peptides with a molecular composition extremely similar to that of collagen. It is produced by partially hydrolyzing collagen. Electrospinning gelatin in water is very difficult, because a polyelectrolyte colloidal sol is produced [56]. Silky gelatin nanofibers have been created using a variety of polar organic solvents, including trifluoroethanol and hexafluoroisopropanol. In addition, a variety of biopolymers have been utilized, including poly(ε-caprolactone) (PCL) [57, 58], silk [59], and carboxymethylcellulose [49].Unlike gelatin and other animal-derived polymers, alginate is a marine-origin polysaccharide that offers distinct advantages, such as metal-chelating ability and ease of gelation, making it attractive for electrospun scaffold fabrication in bone tissue engineering.

#### Alginate

**Fig. 8:**
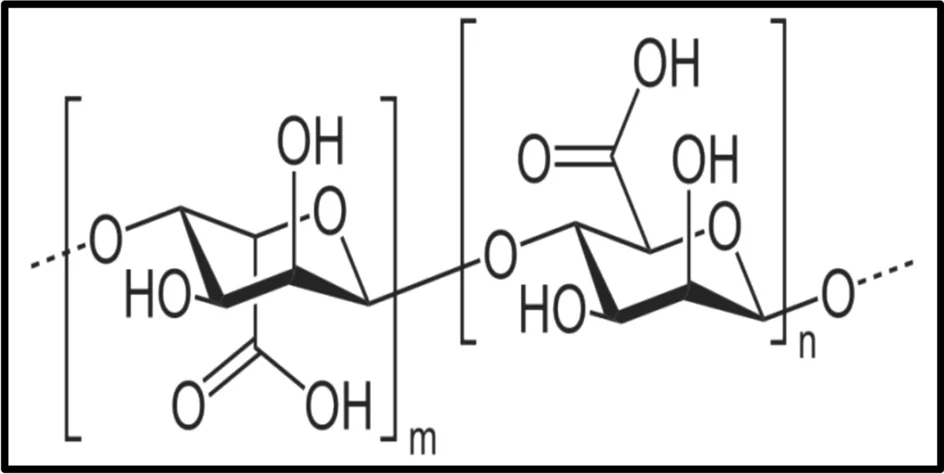
Chemical structure of alginate

Alginate, a naturally occurring anionic polymer often obtained from brown algae (Phaeophyceae), can be treated with aqueous alkali solutions to generate a viscous gum. It is also known as alginic acid. Covalent bonds are used to link it in different patterns or blocks (see Fig. 8). The extensive investigation and application of alginate in biomedicine is driven by its favorable properties, including high biocompatibility, biodegradability, non-immunogenicity, metal-chelating ability, low cost, and natural abundance. As a result of its compatibility with human cells, alginate has been broadly explored for use in tissue engineering applications, including cartilage [60], heart tissue [61], and bone [46]. The first publication of electrospun alginate nanofibers and a discussion of their structural, mechanical, and biological properties was made by Bhattarai et al. [62]. Alginate and poly(ethylene oxide) (PEO) with various eight concentrations have been electrospun, according to Jeong et al. [63]. While alginate is derived from marine sources, hyaluronic acid is a naturally occurring polysaccharide found in all connective tissues of the human body, where it plays a key role in cell signaling, tissue hydration, and extracellular matrix organization, making it particularly suitable for bone tissue engineering applications.

#### Hyaluronic acid

**Fig. 9:**
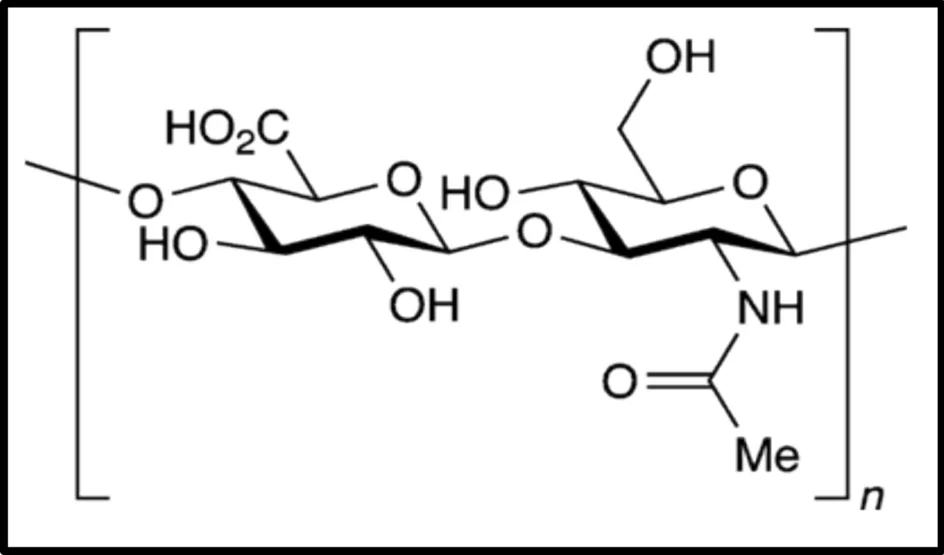
Chemical structure of hyaluronic acid

This polysaccharide is built from repeating disaccharide units of D-N-acetylglucosamine and D-glucuronic acid, which are connected through alternating β-1,4 and β-1,3 glycosidic linkages (see Fig. 9). Hyaluronic acid, which is found in all connective tissues throughout the body, possesses a number of properties, including biocompatibility, excellent viscoelasticity, non-immunogenicity, and water solubility [64]. Another essential quality of hyaluronic acid-supported composites is their capacity for breakdown via enzymatic action.

The rate of enzymatic degradation depends on both the quantity of cleavage sites within the polymer and the concentration of accessible hyaluronidase enzymes in the scaffold’s biological environment. A clinical trial of a synthetic bone graft made of hyaluronic acid and fibroblast growth factor is being conducted to hasten the repair of bone fractures [65]. Nanofibrous scaffolds based on a PLACL–SF–HAP–HA composite were developed to serve as a synthetic analog for the native bone ECM [66]. Human dental pulp stem cell osteogenic adhesion and differentiation were reported to be greatly improved when hyaluronic acid and β-TCP (tricalcium phosphate) were combined [67].In contrast to the polysaccharide-based natural polymers discussed above (chitosan, alginate, and hyaluronic acid), silk fibroin is a fibrous protein biopolymer that offers exceptional mechanical strength and gradual biodegradability, making it uniquely suited for load-bearing bone tissue engineering applications.

#### Silk fibroin (SF)

**Fig. 10:**
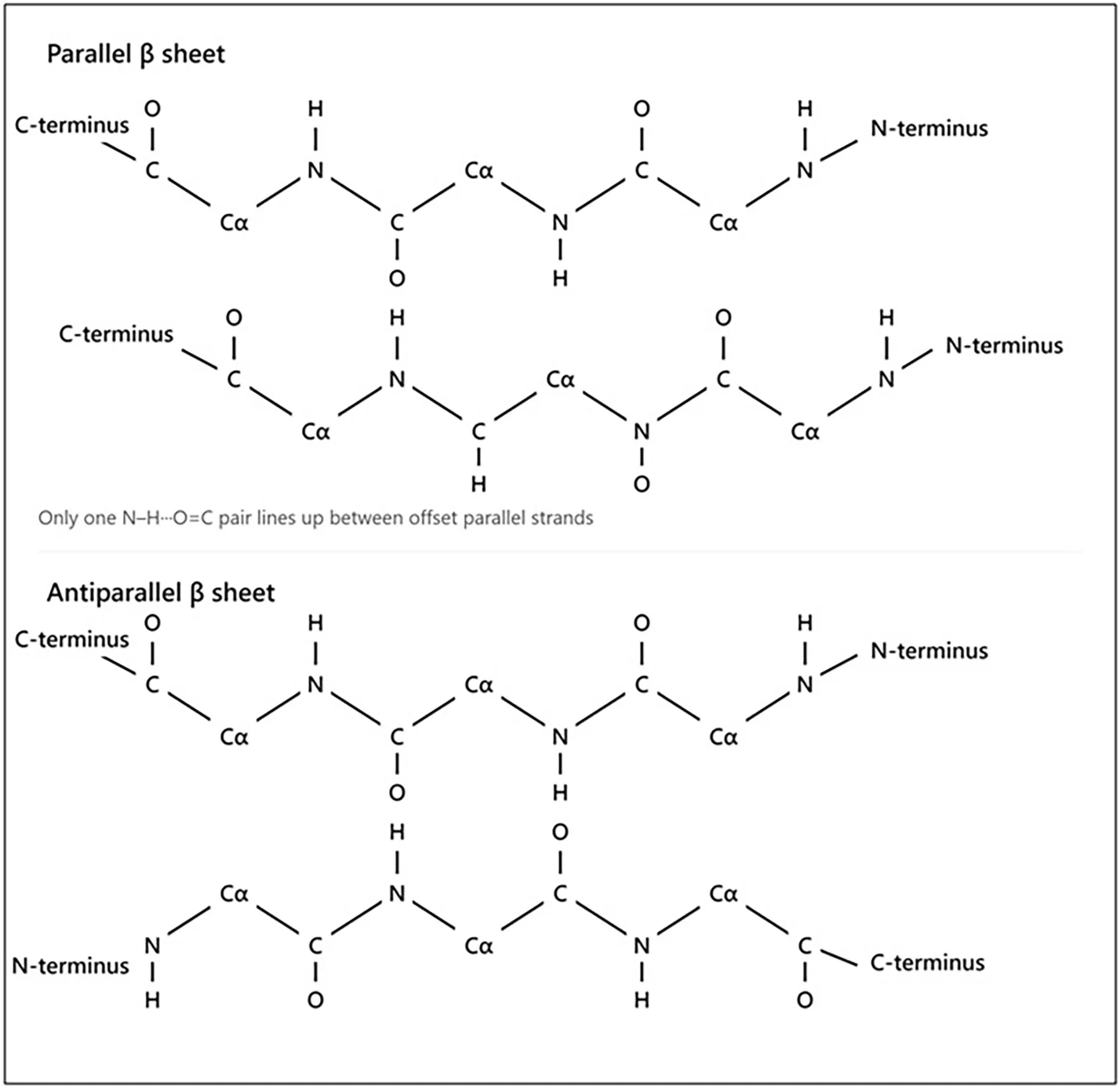
Silk fiber’s parallel and antiparallel β-sheet topologies

Silk fibroin, a fibrous protein biopolymer and a key component of native silkworm filaments, possesses unique physical and chemical properties that make it an attractive material for use in biomaterials [68, 69]. Fibroin, which is the primary structural protein, is coated around silkworm silk fibers that make up cocoons from *Bombyx mori* and serves as a binding agent for the fibers [70]. In both the light and heavy chains, fibers are made up of at least two structural fibroin proteins with molecular weights of 25 kDa and 325 kDa. An antiparallel β-sheet layer in fibroin fibers creates a crystalline region of polypeptide chains, where the hydrophobic chain predominates [71, 72]. Silk fibroin (SF) has been utilized as sutures in medical applications for many years [73], and it is a highly desirable biomaterial for BTE due to its characteristic properties that satisfy many of the requirements for biomaterial composites. Silk fibers demonstrate gradual degradability, genetic customizability of composition and sequence, and permeability to oxygen and water, making them extremely suitable biomaterials for BTE. Moreover, as demonstrated in Fig. 10 [74], their antiparallel β-sheet structure gives silk exceptional tensile and compressive mechanical strength.

Sericin, a protein present in silk fibers, can cause an immunological reaction and inflammation if not properly removed. Therefore, the removal of sericin is a critical step in the processing of silk fibers for biomedical applications. Purified silk fibroin has been shown to have low immunogenicity and induces a foreign body response similar to that of many synthetic materials used as biomaterials. It is, therefore, a viable option for use in biological applications [75]. It has been demonstrated that *Bombyx mori* nonwoven fibroin nets stimulate the growth of osteoblasts and a variety of human cells [76]. Engineered SF matrices have been employed by researchers to create ligaments. Exceedingly porous silk sponges have also been found to effectively promote osteogenesis in MSCs derived from bone marrow. In addition, it has been demonstrated that *Bombyx mori* nonwoven fibroin nets stimulate the growth of osteoblasts and a variety of human cells [77].

Zhang et al. [76] revealed every important element influencing the characteristics of electrospun silk nanofibers. Process-related parameters and substrate-related parameters make up the two kinds of parameters. In recent years, a significant amount of research has been conducted on silk electrospun nanofibers for BTE due to their outstanding mechanical qualities and biodegradability. Chen and co-authors showed how SF/CS composite nanofibers can be used to regenerate bone tissue. According to Zhou et al. [78], electrospun silk nanofibers degrade both in vivo and in vitro. The creation and in vitro decomposition of honeybee silk electrospun nanofibers have been shown by Wittmer et al. [79]. In order to functionalize PHBV nanofibers for BTE, Unalan et al. [80] employed SF as a coating material. A porous.

A composite 3D nanofibrous scaffold was created by electrospinning SF nanofibers, mixing them with starch, and freeze-drying the resulting 3D scaffold. As a final step, CaP was applied to the 3D composite to mimic natural bone ECM. Yang et al. [81] created nHAP/SF composites using a precipitation process. When the amount of apatite was increased to 50 mg, the Young’s modulus of the composite rose to 150 kPa, making it more suited for bone marrow stem cell (BMSC) development. On the surface of SF, Shao et al. [82] used microwave-assisted mineralization to form dense HAP crystals. All of the synthesized biomaterials had typical porosity, with pore widths ranging from 100 µm. Kim HJ et al. [83] utilized an SF membrane adapted with p-benzoyl chloride to biomimetically control nHAP crystal formation in SBF. The results illustrated that when the dosage varied and the temperature increased, the nHAP crystals developed preferentially along the *C*-axis and experienced a morphological shift from spherical to 3D porous structures. Furthermore, the presence of HAP had the potential to promote osteogenic activity. Despite the natural biopolymer SF considerably improving the mechanical strength upon the incorporation of nHAP.

#### Cellulose and its derivatives

**Fig. 11:**
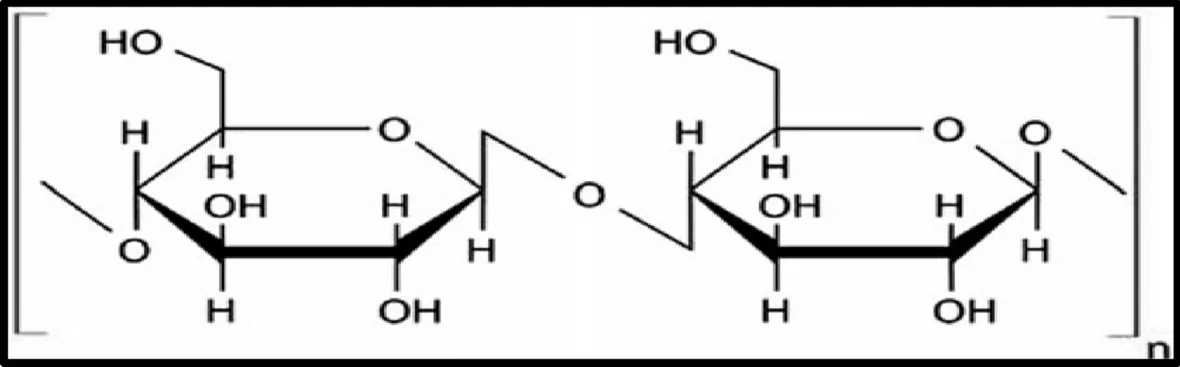
Structure of cellulose

**Fig. 12:**
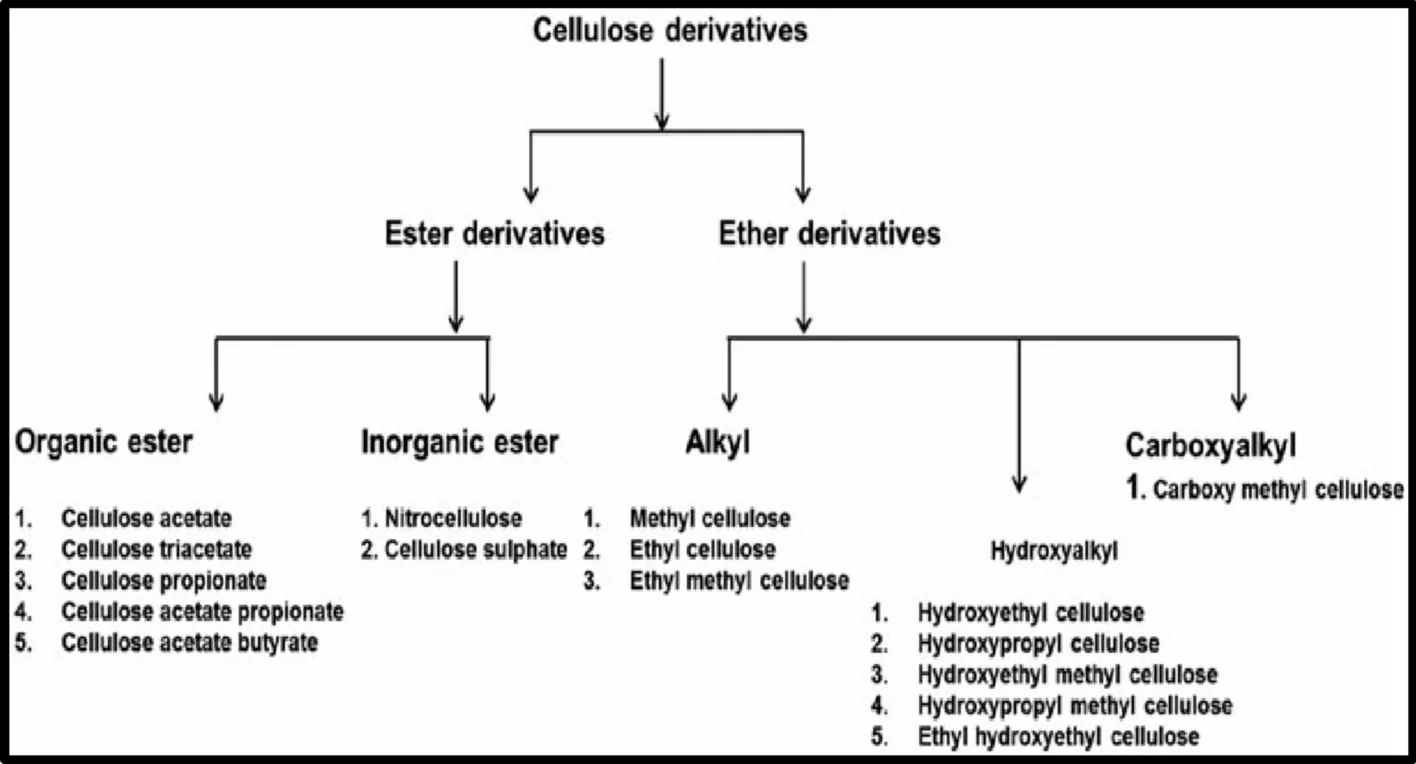
Demonstrate the list of cellulose derivatives

In 1838, a French scientist named Anselme Payen was the first person to extract cellulose from plants. Approximately 5 × 10^11^ tons of cellulose are produced annually, making it the most prevalent natural polymer [84]. The main component of cellulose is cellobiose, a long-chain polymer made up of D-glucose units. Two glucose units are joined by β-1,4 glycosidic linkages to create cellulose, as shown in Fig. 11, and a list of cellulose derivatives is presented in Fig. 12. Cellulose is a valuable biomaterial used for tissue engineering (TE) applications [85]. Cellulose-based biomaterials have been extensively researched in recent years for TE applications due to their excellent biodegradability, low cytotoxicity, and biocompatibility, which are comparable to those of animal and human cells [86]. Cellulose’s linear polymeric chain exhibits exceptionally strong inter- and intramolecular hydrogen bonding. Because of this, it is insoluble in water and most common organic solvents. However, this unique property, along with its non-toxicity, biocompatibility, thermal stability, and mechanical strength, makes it highly appropriate for TE applications.

**Fig. 13:**
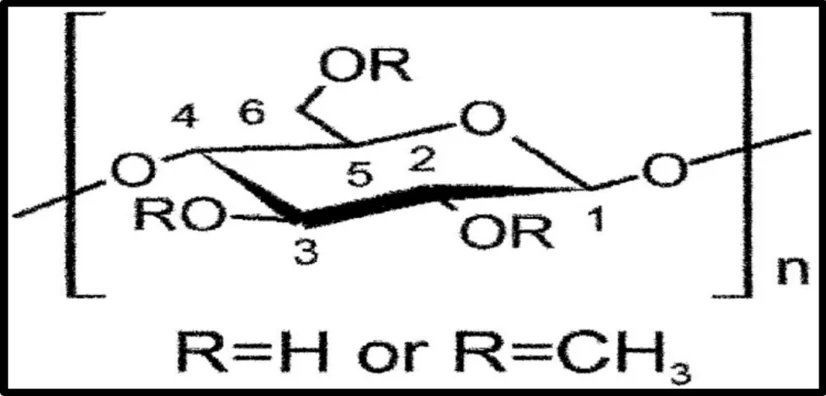
Molecular structure of methylcellulose

Methylcellulose (MC) is a cellulose derivative obtained by introducing –CH₃ groups into the cellulose structure and is water-soluble (Fig. 13). Methylcellulose is a less expensive form of cellulose than collagen. Due to its high water solubility at low temperatures and non-toxicity, methylcellulose, a biodegradable cellulose ether, has been used as a hydrogel solution in cell culture matrices. Its long history of producing gels and fine chemicals for use in paints, ceramics, detergents, medicines, food, polymerization, adhesives, and cosmetics further adds to the interest in MC [87]. Many researchers are interested in it because of its unique properties, such as water solubility, hydrophilicity, non-toxicity, transparency, biodegradability, biocompatibility, and strong film-forming ability. Typically, it has been used in important medical applications, including BTE and drug delivery [88].

To allow for closer examination, aqueous methyl cellulose solutions have been employed to slow the movement of bacterial and protozoan cells. By changing the amount of methyl cellulose present, the solution’s viscosity can be adjusted. Methyl cellulose is also used in research on viral replication in cell culture, as it dissolves in the same nutrient-rich media typically used to grow cells. Research on electrospun nanofiber composites made from cellulose and its derivatives is ongoing. To enhance the mechanical and biological properties of cellulose for tissue engineering applications, it is often combined with a variety of functional synthetic and biopolymers, including collagen, gelatin (GEL), polyvinyl alcohol (PVA), silk fibroin (SF), and polyurethane.

### Synthetic polymers

Biodegradable polymers only remain in the body as long as necessary to complete their task before dissolving naturally without the need for additional surgery. However, difficulties still exist for applications, such as cranial bone grafting, since it is challenging to produce a biomaterial with sufficient flexibility and resilience while maintaining optimal permeability for cell colonization [89]. Because of their superior physical, chemical, and mechanical properties, synthetic polymers are, therefore, seen as promising options for bone substitute materials. The most widely used polymers currently are listed below: methacrylates (e.g., poly(methyl methacrylate), PMMA), silicone (poly(dimethylsiloxane), PDMS), polyolefins (polyethylene, PE; and polypropylene, PP), poly(tetrafluoroethylene) (PTFE), poly(vinyl chloride) (PVC), polyesters (polycarbonates, PC, and poly(ethylene terephthalate), PET), poly(glycolic acid) (PGA), poly(L-lactic acid) (PLLA) poly(D-lactic acid) (PDLA), polydioxanone (PDS), polycaprolactone (PCL), polyethers (polyether ether ketone, PEEK, and polyether sulfone, PES), polyamides (nylon), polyurethanes, semi-synthetic polymers (poly(L-lactic acid), PLLA; poly(β-hydroxyalkanoate), PHA; poly(hydroxybutyrate), PHB; and poly(hydroxybutyrate-co-hydroxyvalerate), PHBV) Because their degradation is relatively consistent across patients and implantation sites, synthetic hydrolytically degrading polymers are favored for several applications, including drug delivery systems and implants. Enzymatic degradation, on the other hand, is the most common mode of biopolymer degradation. In tissue engineering and as an extracellular matrix replacement, this degradation feature is being studied for scaffolds, where it is intended that they vanish with the normal enzymatic turnover of the extracellular matrix. Saturated aliphatic polyesters are an established and popular family of materials in the field of bone tissue engineering [90]. PLA, PGA, PLGA, and several copolymers belong to this family. Importantly, significant progress has been made in developing synthetic biocompatible polymers over the last two decades, with biodegradability being one of the most important properties. Over time, the scaffold should degrade and be replaced by newly regenerated native tissue. Biodegradable polymers are lightweight materials with low elastic moduli ranging between 0.4 and 7 GPa. Because of this, scaffolds composed of biodegradable polymers can be designed to degrade at rates that progressively transfer mechanical load to the regenerating bone. Having established the individual roles of natural polymers and HAP, this section shows their synergistic significance in composite scaffolds. The Comparative performance of natural vs. synthetic vs. composite given in Table 3.

**Table 3:** Comparative performance: natural vs. synthetic vs. composite

S.No	Properties	Natural polymer	Synthetic polymer
1	Cell adhesion & proliferation	Excellent (integrin binding sites)	Poor to moderate (hydrophobic)
2	Mechanical strength	Poor (low modulus, fast creep)	Good to excellent (tunable)
3	Degradation rate	Too fast (days–weeks)	Too slow for PCL (years), adjustable for PLGA
4	Osteogenic differentiation	Moderate (needs mineral)	Low without bioactive cues
5	Electrospinnability	Difficult (needs blending or crosslinking)	Excellent

The critical gap of polymer composites is mineral distribution within nanofibers vs. on the surface. Most studies show mineral on fiber surfaces, not inside. However, intrafibrillar mineralization (mimicking natural bone) is superior for mechanical performance. The review should note that only a handful of recent studies using polymer-induced liquid precursor (PILP) or non-classical nucleation pathways have achieved true intrafibrillar mineralization in electrospun composites.

## Significance of natural polymer and HAP

Both hydroxyapatite (HAP) and natural polymers are significant substances in the biomaterials sector, yet they serve distinct purposes. Natural polymers derived from biological sources—such as collagen, silk fibroin, chitosan, and hyaluronic acid—are biocompatible and biodegradable. They have numerous biological applications, including wound dressing, drug delivery, and tissue engineering. Hydroxyapatite, on the other hand, is a synthetic material that closely resembles the mineral component of natural bone. Due to its excellent osteoconductive properties and biocompatibility, it is ideal for bone augmentation and regeneration.

Recent developments in bone-regeneration biomaterials focus on the potential of calcium phosphates (CaPs), primarily HAP, to induce bone regeneration. Recently, the osteogenic characteristics of CaP ceramics have frequently been enhanced by the addition of growth factors, such as BMP-2 (bone morphogenetic protein-2). However, growth factors may promote ectopic or undesired bone formation, raising concerns regarding their safety. As a result, several studies have examined alternative pathways to enhance osteogenic properties, specifically through the stimulation of bone growth. Introducing elements, such as Ag⁺, Cu^2^⁺, Mg^2^⁺, Na⁺, Zn^2^⁺, Si^2^⁺, and Sr^2^⁺ into CaP, mainly hydroxyapatite, may produce the desired results [91, 92]. Numerous studies on substituted HAP have gradually increased over the past three decades. Less than 2% of all studies up to 2005 consisted of substituted HAP, normalized to the total number of HAP publications. Since then, the percentage of publications on the topic of substituted HAP has increased significantly, rising from 3.3% in 2010 to 6.3% in 2015, and subsequently to 9.5% in 2019. The number of elements being investigated as potential HAP substituents has increased alongside the quantity of published studies [93].

It is vital to examine the location of the HAP structure in order to gain a thorough understanding of the replacement mechanism and how it impacts bone formation and the properties of HAP. Because they are biocompatible, biodegradable, and can replicate the extracellular matrix (ECM) of natural tissues, natural polymers are important for tissue engineering and regenerative medicine. HAP, on the other hand, is a substance that is ideal for bone regeneration, since it closely resembles the mineral component of natural bone. Therefore, both natural polymers and HAP have their unique advantages and applications in the field of biomaterials.

HAP has several significant applications in the field of biomaterials, including:

(i) *Bone regeneration and augmentation* HAP has excellent osteoconductive properties, meaning that it can stimulate bone growth and regenerate bone defects. It can be applied to bone defects to heal and regenerate them, in procedures, such as spinal fusion, craniofacial surgery, and joint replacement.
(ii) *Coating for medical implants* HAP can be coated onto medical implants, such as dental implants, to encourage osseointegration—the process by which the implant fuses with the surrounding bone tissue. This enhances the stability and longevity of the implant.
(iii) *Drug delivery* HAP has the potential to serve as a drug delivery vehicle. For targeted therapy, it can be loaded with medications, growth factors, or other therapeutic agents and delivered to the site of injury or disease.
(iv) *Tissue engineering* Scaffolds for tissue engineering can be made by combining HAP with other biomaterials, such as synthetic or natural polymers. While the other materials supply the required mechanical and biological properties, the HAP component provides the mineral component necessary for bone tissue engineering.

All things considered, HAP has a significant impact on the biomaterials industry and has numerous applications in drug delivery, medical devices, and regenerative medicine. Its osteoconductive properties and biocompatibility make it an ideal material for bone augmentation and regeneration, and its versatility and adjustable characteristics make it beneficial for a range of biomedical applications.

## Fabrication of biocomposites by different techniques

A biocomposite acts as a matrix for cell adhesion, differentiation, and proliferation, and also allows cell infiltration into the scaffold, which helps maintain the predetermined tissue structure. Surface topography at the micro/nanoscale, as well as mechanical stiffness, can push stem cells toward the osteogenic lineage. Therefore, fine-tuning of biocomposites can be performed to provide a physical environment for osteogenic stimulation. To achieve this, bone tissue composites have been created using a number of techniques, including electrospinning, solvent casting, phase separation, and self-assembly.

### Self-assembly

**Fig. 14:**
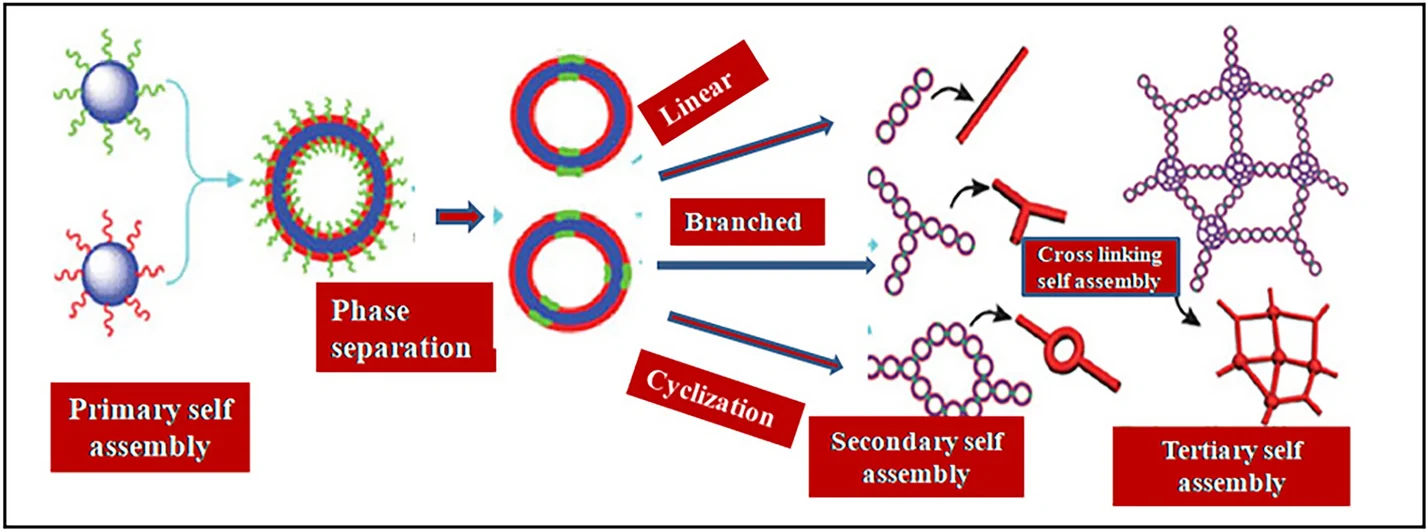
Flow chart illustrating the self-assembly of polymers

The self-assembly process (Fig. 14) occurs when separate constituents organize themselves into a pattern or structure through pre-programmed non-covalent interactions [12]. The fundamental forces underlying this process are the intermolecular interactions produced between the several components involved in self-assembly, such as hydrogen bonds, hydrophobic interactions, and van der Waals contacts [94]. Self-assembly occurs frequently in nature; however, it can only happen in certain polymer topologies. In addition, this method cannot be employed to fabricate bone tissue engineering (BTE) composites on a large scale due to its complexity and low fiber yield [95].

### Phase separation method

The following steps were adopted in phase separation methods. The polymer solution was split into two phases by liquid–liquid phase separation, one of which was polymer-rich, whereas the other was polymer-poor. The second phase dissolved, while the first phase hardened, resulting in an exceptionally porous network [12]. Varying process parameters such as polymer content, temperature, and quenching rate influenced the resultant composite’s micro- and macrostructure. The process used low temperatures, which facilitated the incorporation of bioactive substances into the structure. Nanoscale fibrous structures that resemble the architecture of the extracellular matrix and offer an ideal environment for cell attachment and activity are produced using phase separation technology. Nevertheless, this method is highly limited in that it could only be utilized with a certain type of polymer [12]. One of the major disadvantages of this method is the physical separation of the phases; it is difficult to obtain a single phase.

### Freeze-drying method

Polymeric porous composites can also be produced by freeze drying. The polymer solution is cooled until all of its constituents have frozen, and ice crystals form from the solvent, compelling polymer molecules to form interstitial gaps. The solvent is removed in the second phase by applying a pressure lower than the vapor pressure of the frozen solvent at the equilibrium point. A dry polymer composite with an interconnected porous microstructure is created when the solvent is completely sublimated. The porosity of the composites is controlled by the polymer solution concentration, while freezing temperatures affect the pore size distribution. This method is used to dry biological material in order to preserve its bioactivity while also producing porous scaffolds. One of the main disadvantages of this method is that it takes a long duration to produce the scaffolds, and it is a high-cost method.

### Casting method

**Fig. 15:**
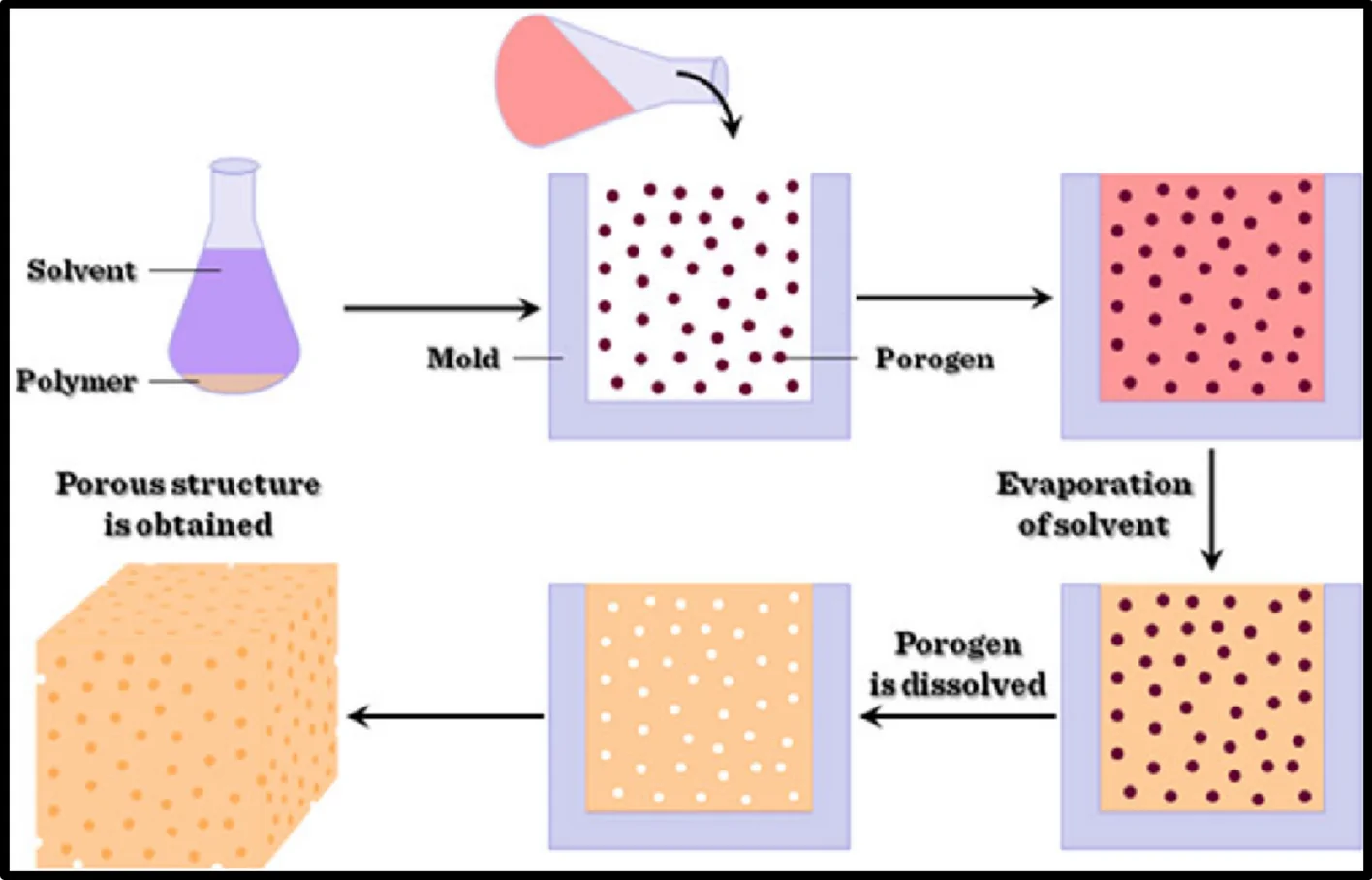
Casting process

In the casting method (Fig. 15), the polymer solution is regularly blended with salt particles of different sizes, and then the solvent is evaporated, leaving behind a polymer matrix containing salt particles. When the matrix is immersed in water, the salt is leached out and forms a porous structure. This method can result in very porous scaffolds with average pore diameters of up to 500 µm and porosity values of up to 93%. The drawback of this technology is that it can only produce thin membranes up to 3 mm thick. The use of organic solvents, which prevents the addition of medicinal substances to the membrane during manufacture, is another downside of this method.

### E-spin (Electrospinning)

Electrospinning is a versatile technique that has recently become popular for the development of nanofibers [96]. Between 1600 and 1995, various developments and discoveries were made in this field. Since the 1980s, and more recently, the electrospinning technique has received increased attention, most likely due to growing public interest in nanotechnology [97]. The technique was advanced by the work of Jirsak and his colleagues, who introduced a notable electrospinning method in 2009 at the Technical University of Liberec in the Czech Republic [98]. Significant advances in electrospinning over the last decade have enabled the production of biocomposites made from various materials. As a result, this approach has grown in prominence in tissue engineering research. The major advantage of nano-porous electrospun composites is that they provide a biomimetic extracellular matrix pattern and produce crystals that create an overall osteoconductive and osteoinductive environment. The porous structure of electrospun composites can enhance bone ingrowth, improve healing, and allow faster revascularization.

#### Principle of E-spin process

**Fig. 16:**
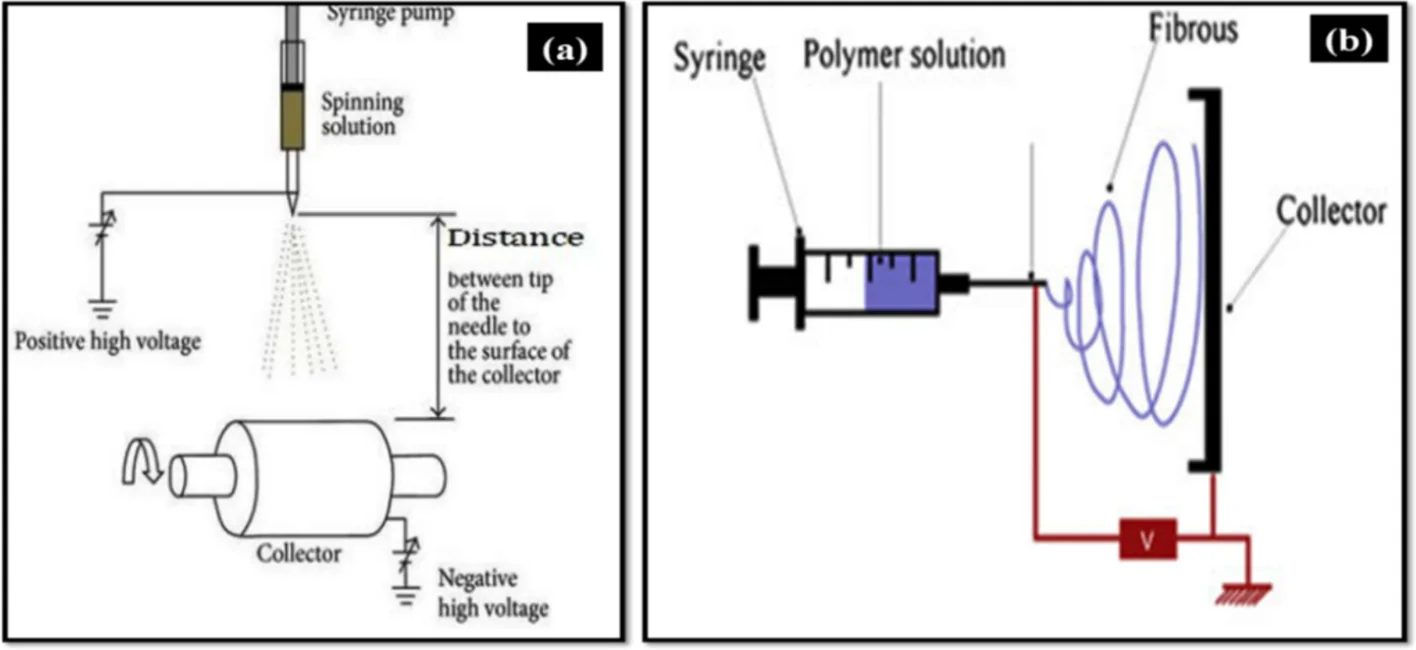
Schematic illustration of E-spin system: **a** vertical system; **b** horizontal system

The polymer solution is transformed into a charged liquid jet when subjected to high voltage during the electrospinning procedure to produce nanofibers [99]. The standard electrospinning method is a complex, physics-governed procedure. The classic approach involves dissolving a polymer in a suitable solvent, loading it into a syringe fitted with a metal needle connected to a syringe pump (see Fig. 16), and controlling the polymer solution’s flow rate.

**Fig. 17:**
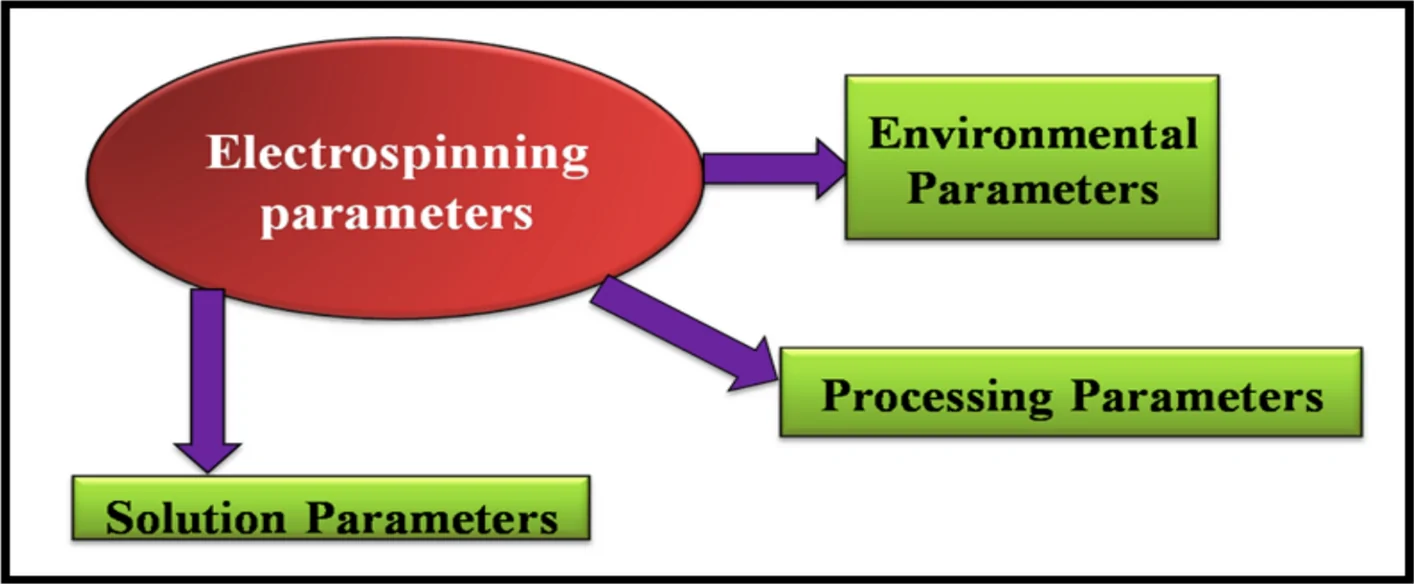
Electrospinning parameters

The production of charged fluid jets is influenced by voltage. At low voltage, the polymer solution flows down the needle’s surface, whereas high voltage induces the formation of a cone, known as a Taylor cone. A jet of charged fluid is emitted from the tip of the Taylor cone and accelerates toward the collector after the intensity of the electric field surpasses the surface tension (Fig. 16). Because the jet is charged, an electric field can be used to alter its path before it dries as nonwoven fibers on the collector. The fiber diameter decreases from the micrometer scale to tens of nanometers as a result of the continual stretching and whipping of the jet en route to the collector. The polymer solvent evaporates on the collector to produce dry nanofibers. The shape and structure of electrospun nanofiber composites are influenced by a variety of factors, such as (i) polymer solution parameters, (ii) processing parameters, and (iii) environmental parameters, as presented in Fig. 17.

#### Control over the morphology and diameter of nanofibers

The shape of electrospun nanofibers is influenced by polymer solution properties, processing parameters, and environmental factors. Regarding polymer solution characteristics, the primary variables affecting nanofiber morphology are viscosity, conductivity, surface tension, concentration, and molecular weight. Fiber diameter tends to increase as polymer viscosity and concentration increase. Conversely, when the solution’s conductivity increases, the fiber diameter decreases. In contrast, the solution’s surface tension has no significant effect on fiber diameter or morphology.

Applied voltage, flow rate, and collector distance are important processing parameters. An increase in applied voltage enhances the electrostatic force, potentially resulting in the production of nanofibers with smaller diameters [100]. The distance between the needle and the collector influences the applied voltage and electrostatic field strength, which in turn affects the extent to which the polymer jet expands. Sufficient space must also be provided for solvent evaporation. Fiber orientation is influenced by the shape of the collector plate: aligned or partially aligned nanofibers are typically collected on rotating drum collectors, whereas randomly oriented fibers are frequently collected on flat collectors [101].

Temperature and humidity levels inside the electrospinning setup affect the environment and, consequently, the fiber diameter. Higher temperatures lead to a decrease in fiber diameter, while lower humidity allows for faster flow rates, which ultimately minimizes bead formation. The high surface area-to-volume ratio of nanofibers is a key property that makes them highly advantageous for use in tissue engineering.

#### Electrospun polymers for BTE

**Fig. 18:**
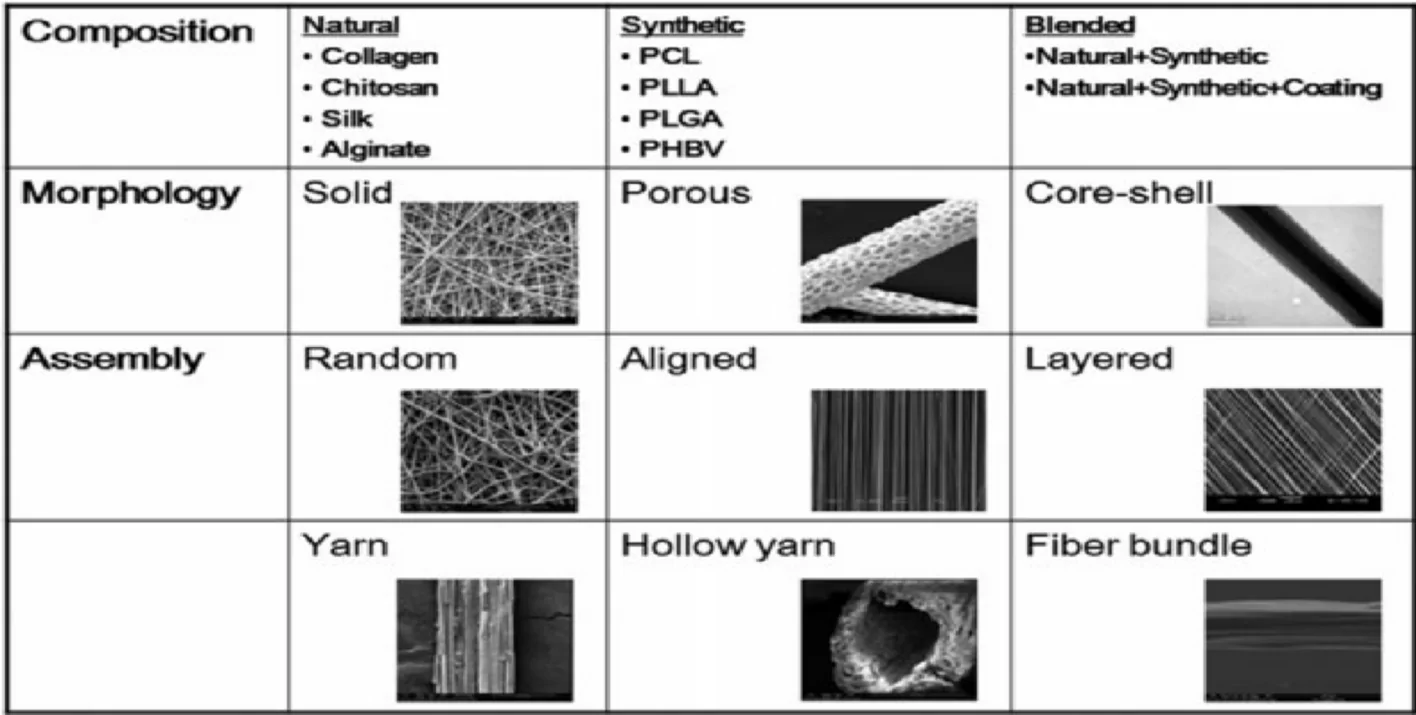
Topography of different electrospun nanofibrous structures

Electrospinning is a manufacturing process that produces nanofibers with controlled diameters and unique structures; however, the material used depends on the fibers’ final application. Over the last two decades, significant interest has developed in electrospinning technology and the nanofibrous matrices it produces. This attention stems from their structural resemblance to the native extracellular matrix (ECM) and the relative accessibility of the electrospinning process, as illustrated in Fig. 18 [12]. Because certain polymers are chemically or structurally similar to human tissues, they can be used for cell adhesion to encourage cell proliferation. Some polymers can, therefore, serve as advantageous biomaterials when processed via electrospinning. Moreover, one of the most significant factors to consider when selecting a material for specific biological applications is its biodegradability. The electrospinning process allows precise control over fiber morphology, porosity, and mechanical properties. However, to be effective for BTE, the resulting scaffolds must also satisfy several critical biomaterial requirements, discussed next.

## Properties required for biomaterial to mimic ECM

The following characteristics are crucial, for the creation of perfect biomaterial composites that resemble the ECM of real bone.

### Biocompatibility nature

Any form of bone composite must satisfy the crucial condition of biocompatibility. This can be described as the scaffold’s capacity to maintain normal cellular activity without negatively influencing the surrounding bone tissue engineering (BTE) environment [102]. The ideal composites should be osteoconductive, allowing bone cells to adhere, migrate, proliferate, differentiate, and ultimately form the extracellular matrix (ECM).

### Biodegradability nature

The biodegradability of synthetic biomaterials is critical. The ideal bone composite should degrade over time at a controlled rate to allow for the formation of new bone tissue. The degradation performance of a bone composite varies depending on its intended application; for example, a degradation period of less than 9 months is desirable for spinal fusion.

### Mechanical property

A successful bone-regeneration composite must possess superior stress- and strain-bearing capabilities while also matching the mechanical properties of the surrounding tissues [28]. Several critical mechanical properties are commonly measured, including tensile strength, elastic modulus, compressive strength, fracture toughness, and Young’s modulus. As a result, it is critical to find the optimal balance, where the mechanical characteristics of the scaffolds are comparable to those of natural bone.

### Cell adhesion and signaling

The material needs to support cell attachment and provide the biochemical signals required to drive cellular proliferation and differentiation.

### Porosity measurement

For cells to survive, interconnected porosity is required in bone composites to replicate the pore size of the natural extracellular matrix and to enable nutrition and oxygen transport. According to Woodard et al., the pore size of the composites ranges from 100 to 350 µm. While excessively porous composites are detrimental to mechanical strength and cell adhesion, an appropriate porous structure enhances cellular ECM production, which is crucial for bone regeneration. For this reason, biocomposites with suitable porosity provide a more dependable solution for bone repair.

### Bioactivity

The implant must be biocompatible and actively encourage tissue repair, potentially through the controlled release of biochemical-like growth factors.

### Surface chemistry

Optimizing surface chemistry is essential for directing cellular behaviors such as adhesion, differentiation and proliferation toward a targeted therapeutic goal.

## Bioactivity of electrospun nanofibers

Biomineralization is a biologically controlled process, wherein organisms precipitate inorganic minerals from their environment, systematically integrating them into complex and functional structures. To promote cell adhesion and development, electrospun composites are designed to function like a temporary, synthetic extracellular matrix (ECM) that resembles that of natural bone. The primary organic and inorganic components of the native bone ECM are nanofibrillar collagen and nanocrystalline hydroxyapatite, respectively. They aid in the migration of carbohydrates, nucleic acids, lipids, and proteins to construct the ECM [103]. Osteoconductivity depends on the topographical characteristics of composite nanostructures, because bone cells are more likely to attach, develop, differentiate, proliferate, and create ECM as a result of interactions at the nanoscale.

Similar to the nanostructure of collagen fibrils, electrospinning fabricates composites featuring a nanofibrous, highly porous architecture with extensive interconnectivity. To mimic the nanostructure topography of natural bone, recent strategies have focused on incorporating nano-hydroxyapatite (nHA) particles into electrospun nanofibers or inducing surface mineralization, enabling the fabrication of biomineralized nanofibrous scaffolds. The bioactivity of the material can be assessed by immersing it in two different ionic solutions that simulate bodily fluids; an alternative calcium phosphate soaking technique can also be used to achieve surface mineralization.

### Simulated body fluid (SBF)

**Fig. 19:**
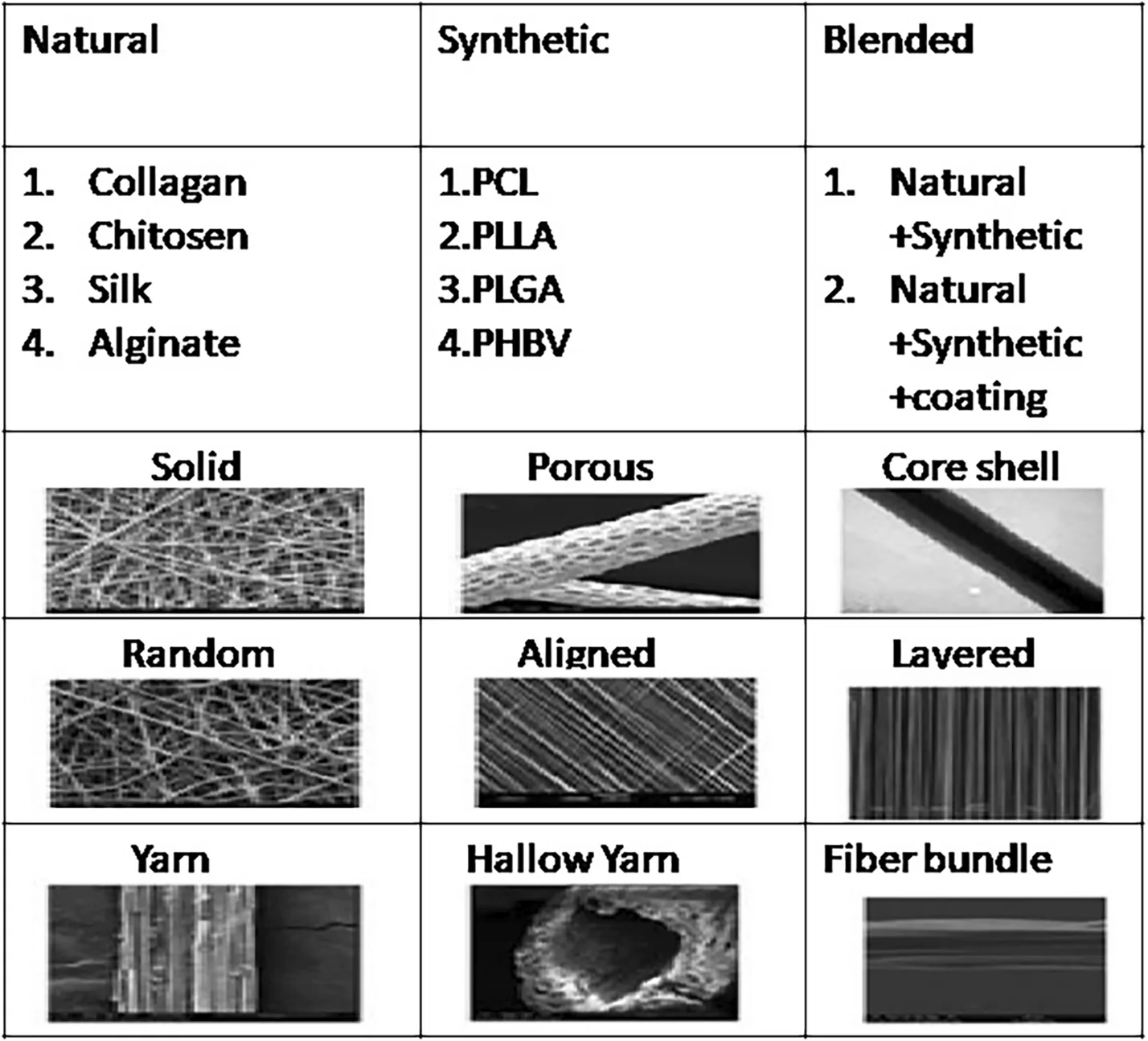
Schematic presentation of the mechanism for the formation of bone-like apatite formation in SBF [12]

For the first time in 1980, Ogino et al. [104] showed that a CaP film could develop in vitro in a buffer solution containing Tris buffer at pH 7.4. The ion concentrations of Ca^2^⁺, Na⁺, HPO₄^2^⁻, Cl⁻, Mg^2^⁺, K⁺, and SO₄^2^⁻ in the traditional simulated body fluid (SBF) are such that the electrolyte profile of this solution is physiologically balanced to closely resemble human blood plasma. However, it is distinct due to the intentional omission of bicarbonate ions (HCO₃⁻), which are replaced by an alternative buffering agent [105]. According to the existing literature, the formation of bone-like apatite on the surface of a biomaterial is a prerequisite for its successful implantation in a living organism. The ionic composition of SBF is engineered to closely match the concentrations found in human blood plasma. This similarity creates a biomimetic environment that promotes the nucleation and growth of bone-like apatite, a calcium phosphate mineral, on material surfaces. Figure 19 illustrates the proposed mechanism for this process.

The mineralization of nanofibrous scaffolds by SBF is generally recognized to produce bone-like apatite, which encourages bone cell attachment. Research by Chahal et al. [106] demonstrated that incorporating bone-like apatite into hydroxyethyl cellulose/polyvinyl alcohol (HEC/PVA) biocomposites improved the material’s mechanical strength and enhanced its functionality by supporting osteoblast growth.

## Interface adherence and biocompatibility

Numerous definitions have been offered for the word biocompatibility. Materials that produced the fewest biological reactions were formerly considered biocompatible. A biomaterial is considered biocompatible if it can fulfill its intended therapeutic purpose without causing harmful local or systemic reactions in the recipient. In that specific scenario, it should maximize the therapeutic effect and induce the most advantageous cellular or tissue response. A key property of a tissue engineering scaffold is biocompatibility, meaning it must serve as a supportive framework that enables normal cell behavior and facilitates crucial communication through mechanical and molecular signaling [107].

The compatibility of scaffolds with biological systems is primarily influenced by their chemical composition, structural design, and morphology, which in turn are affected by factors, such as sterilization methods, fabrication processes, and polymer synthesis techniques. In recent years, numerous biodegradable polymers—including PLA, PGA, PLGA, PDO, PTMC, and others—have been extensively explored and applied in various medical fields because of their excellent biocompatibility [108, 109]. The interaction of mammalian cells with polymeric materials—particularly their adhesion and growth—is strongly influenced by surface properties, such as wettability, overall chemical composition, balance between hydrophilicity and hydrophobicity, surface charge and its distribution, as well as surface roughness and mechanical stiffness. Various surface treatments can be applied to surfaces that come into contact with living tissue to increase their biocompatibility. A coating can be used to keep unwanted residues or additives out, and a selectively permeable surface can be used to control absorption and/or excretion. Recent studies have utilized surface modification techniques—including ammonia plasma treatment, polystyrene oxidation, and acetone plasma deposition—to alter the chemical and physical properties of polymeric biomaterials. These treatments are applied to directly influence cellular adherence and proliferation.

For biomaterials to provide a suitable environment for tissue repair and regeneration, their design must capture the complex interplay of physical, chemical, and biological features inherent to the natural extracellular matrix. The combination of polymers and hydroxyapatite (HAp) is extensively researched for bone tissue engineering, owing to its significant potential in the field.

## Hydroxyapatite with polymer composite

A detailed literature survey on various materials and nanocomposites in biomedical applications, in particular for bone defect applications are as follows.

The Silk fiber/carboxymethyl chitosan was reported by He et al. in [110]. They mixed the films with a 5–10% CMCS concentration, which demonstrated a decreased ability for absorbing moisture, swelling, and water solubility. When CMCS was added to blend films in amounts more than 15%, these qualities improved. It was used for swelling measurement applications. Rong et al. [111] made the Silk Fibroin/Chitosan composites. Evaluation of the nanofiber-reinforced aerogel composite confirmed its biocompatibility with MC3T3-E1 cells in vitro. Furthermore, the material promotes osteogenic differentiation, a function facilitated by its rough surface topography, high mechanical strength, and carefully regulated pore architecture. Narayana et al. [112] synthesized the Silk Fibroin/Chitosan materials. These promising results enhance our understanding of blood coagulation and re-epithelialization, indicating that Silk Fibroin/Chitosan composites are a strong candidate for developing advanced wound-healing devices that benefit both patients and healthcare systems. Kundu et al. [113] made Silk Fibroin/Sodium Carboxymethylcellulose and were determined the potential substratum for maintaining cell proliferation and adhesion and tissue engineering applications.

According to Singh et al. [114], composite nanofibers composed of silk fibroin (SF) and carboxymethylcellulose (CMC) were fabricated for bone tissue engineering applications. The study aimed to evaluate the capacity of these SF/CMC composites to induce the nucleation of bioactive nano-sized calcium phosphate (Ca/P) crystals via a biomineralization process.

A study by Yooyod et al. [115] fabricated a novel biomaterial scaffold composed of silk sericin and poly (vinyl alcohol). The research highlighted its potential as a foundational technology for regenerating skin and other tissues. According to He et al. [116], a novel Ag-NPs–sericin/PVA film demonstrates considerable promise for use in biomedical materials, including applications in wound dressings and skin tissue engineering. In their 2018 study, Sheik and colleagues [117] fabricated composite poly(vinyl alcohol) (PVA) films reinforced with chitosan-grafted silk fibers. This was achieved by first modifying the silk fiber surface with an anhydride, followed by grafting the polysaccharide chitosan onto the fiber via an acyl bridge. Ojah et al. [118] reported on the development of AMOX–BMSF/PVA nanofibers, a surface-modified version of silk/PVA nanofibers. Their research indicated that this mechanically enhanced material is a promising candidate for wound dressing applications, particularly for combating skin and wound infections. According to Candi et al. [119], a triboelectric nanogenerator fabricated from a poly (vinyl alcohol)-silk fibroin composite demonstrated several advantageous properties, including thermal stability, high transparency, and favorable mechanical resistance with flexibility. The study concluded that this prototype shows significant potential for the large-scale manufacturing of bio-based energy harvesters. In their 2018 study, Sheik and colleagues [120] produced PVA/PVP hybrid composites reinforced with different weight percentages of silk fibroin (SF) via a casting technique. The research found that the composite films exhibited strong mechanical properties, including tensile strength and Young’s modulus that increased with fiber content, though elongation at break was inversely affected. Their favorable biodegradability makes them suitable for use as wound dressings.

According to Xu et al. [121], porous scaffolds composed of silver, strontium hydroxyapatite, and chitosan (Ag–SrHAP/CS) were prepared. The study demonstrated that this construct possesses a combination of excellent biocompatibility, osteoinductive potential, and antibacterial properties, making it a highly promising candidate for applications in bone defect repair and infection prevention. Anjaneyulu et al. [122] reported that composite nanofibers composed of silver-doped hydroxyapatite and polyvinyl alcohol (Ag-HA/PVA) functioned as a promising scaffold material for use in tissue engineering. In their 2021 study, Sofi et al. [123] reported on the development of a tissue engineering scaffold based on the incorporation of hydroxyapatite (HAp) and silver (Ag) nanoparticles. Ofudje et al. [124] developed and characterized a Zn-doped hydroxyapatite scaffold for its bioactivity and antibacterial efficacy. Similarly, Ran et al. [125] created a multi-doped (Mg, Zn, Sr, Si) nano-hydroxyapatite/chitosan composite intended for use as a load-bearing bone implant. Several studies have developed various zinc-hydroxyapatite composites to promote bone regeneration. Manivannan et al. synthesized a beeswax–chitosan/Zn-HAp biocomposite [126]. In a rabbit model over 2 months, BMP‑2‑loaded Zn‑HAp and HAp/collagen (HAC) exhibited no inflammation and formed a well‑differentiated bone matrix, with Zn‑HAp inducing more new bone (50%) than HAC (27%) [127].The amorphous phase of HAC impaired bone formation. Begam et al. demonstrated that Zn-HAp/collagen composites, especially when combined with BMP-2, accelerate in vivo osseointegration. These composite scaffolds are effective as they mimic the native extracellular matrix, providing an ideal surface for osteoblast activity [128, 129], Table 4. Summary of electrospun composite scaffolds for bone tissue engineering: materials, properties, and bioactivity.

**Table 4:** Composition, mechanical performance, and bioactivity of electrospun composite scaffolds for bone tissue engineering

S.No	Material	Polymer	HAP/Dopant	Young’s Modulus (GPa)	Tensile strength (MPa)	Cell viability (%)	Bioactivity outcome	Limitation	References
1	nHAP/SF	Natural (Silk)	nHAP (50 mg)	0.15	–	Not reported	Supports BMSC development	Low modulus, not load-bearing	[81]
2	Ag–SrHAP/CS	Natural (Chitosan)	Ag⁺, Sr^2^⁺	–	–	Excellent (qualitative)	Osteoinductive + antibacterial	No quantitative mechanical data	[121]
3	PCL/GE/HAP 50%	Synthetic (PCL)	HAP (50% w/v)	–	–	> 85%	Increased bone formation in vivo (6 weeks)	High HAP content increases brittleness	[149]
4	Sr/Zn-nHAP/Col/PLGA	Synthetic (PLGA)	Sr^2^⁺, Zn^2^⁺ (4%)	9.91	–	–	Sustained ion release, apatite formation	Hardness (0.30 GPa) may limit ductility	[145]
5	PVA/tri-metal HAP	Synthetic (PVA)	Cu^2^⁺, Mn^2^⁺, Sr^2^⁺ (40%)	–	5.67	74% (at 10 μg/mL)	Antibacterial (S. aureus 20.8 mm, E. coli 21.8 mm)	Moderate cell viability, IC50 21.66 μg/mL	[153]
6	SF/GelMA/nHA 7:2:1	Natural/Synthetic blend	nHA (in situ)	–	–	Excellent	110% elongation, apatite formation in 3 days	Complex fabrication, multi-step process	[152]
7	COL/SF/CaO–SiO₂	Natural blend	Bioactive glass (20 ± 5 nm)	–	–	–	Enhanced vascularization + osteogenesis in osteoporotic rat model	No standalone HAP, complex ternary system	[133]
8	PHB/Nb₂O₅	Synthetic (PHB)	Nb₂O₅ (6%)	–	3 × increase over pure PHB	Non-toxic	Upregulated osteogenic genes, peak ALP activity	Nb₂O₅ is not HAP; different reinforcement mechanisms	[136]

## Recent advancement in biomimetic double-layered electrospun nanofibrous

A double-layered biomimetic nanofibrous scaffold with TGF-β3 and mussel adhesive protein coating was developed to improve tendon-bone healing in rotator cuff repair. It promoted cell adhesion, stem cell recruitment, and chondrogenic differentiation via PI3K–Akt signaling, enhancing cartilage regeneration, enthesis structure, and biomechanical properties in a rat model [130]. Inspired by reptile eggshells, a modular keratin-based composite was engineered with customizable vapor sorption. Key biomimetic components include fibrous membranes, protein matrix, calcium carbonate, and lipid coating. Adjusting CaCO₃ content and organic/lipid layers enabled fine-tuned absorption, replicating natural eggshell behavior for applications in wound dressings, scaffolds, packaging, and filtration [131]. Electrospun biomimetic scaffolds were developed using chitosan with alginate or hyaluronic acid (HA), plus collagen for bioactivity. Collagen-enhanced scaffolds improved thermal stability and swelling control, while HA-based ones offered finer fibers and larger pores but lower strength. Cell viability reached 85% with higher collagen, whereas high HA content reduced it to 71%. [132]. A multifunctional bone-like nanofiber scaffold (COL/SF/CaO–SiO₂) was developed, mimicking native bone matrix structure. Bioactive glass nanoparticles (20 ± 5 nm) anchored on collagen–silk fibroin fibers (115 ± 10 nm) enhanced osteogenic activity. In an osteoporotic rat model, the scaffold significantly promoted bone regeneration and vascularization, offering a promising strategy for complex bone repair [133]. A novel cardiac scaffold was developed by blending elastic poly(glycerol sebacate) with conductive polyaniline (PANI) using PVA as a carrier. The aligned or random nanofibers offered tunable conductivity, elastic modulus (0.65–2.18 MPa, mimicking myocardium), slower degradation, good hemocompatibility, and promoted H9c2 cell adhesion/proliferation. In vivo tests confirmed good biocompatibility for cardiac tissue engineering [134]. This study investigates vibrational behavior in electrospun enthesis-mimicking scaffolds. PLLA/collagen type-I bundles replicated fibrocartilage, junction, and tendon regions, while PEOT–PBT bundles represented tendon only. PLLA/Coll scaffolds showed geometry-dependent dominant vibrations up to 4.2 ± 0.9 Hz and spatial gradients in modulus and energy dissipation, unlike PEOT–PBT, which exhibited higher vibration attenuation (0.539 ± 0.063 Hz). These findings aid design of mechanically gradient scaffolds for tendon/ligament-bone interface repair [135]. A biomimetic electrospun membrane based on polyhydroxybutyrate (PHB) with niobium oxide (Nb₂O₅) was developed for bone tissue engineering. Nb₂O₅ reduced fiber diameter, decreased crystallinity, and improved tensile strength (threefold increase). Membranes showed no toxicity, enhanced osteoinductive potential, and upregulated osteogenic gene expression in hBMSCs, with peak mineralization and ALP activity at 6% Nb₂O₅ content [136]. A Janus piezoelectric nanofibrous scaffold was developed for rotator cuff repair, featuring aligned PLLA/ZnO (tenogenic) and random PLLA/BTO (osteogenic) layers. It provided biomimetic fiber orientation, high tensile and suture retention strength, and exercise-driven electrical stimulation. In a rat model, the scaffold promoted simultaneous tendon-to-bone healing, demonstrating a promising strategy for sports medicine applications [137]. Mineralized polymeric/carbon nanofibers mimic bone ECM, offer high surface area, and enhance bone healing. This review covers fabrication methods and bone tissue engineering applications [138]. Table 5 shows the summary of electrospun nanofibrous scaffolds for bone tissue engineering applications. Electrospun nanofibers are widely explored for bone tissue engineering due to their ECM-mimicking structure, high porosity, and tunable properties. Key materials include natural polymers (collagen, silk fibroin, gelatin), synthetic polymers (PCL, PLGA, PU, PVA), and bioactive ceramics (hydroxyapatite, bioactive glass). Doping with ions (Sr^2^⁺, Zn^2^⁺, Cu^2^⁺) or incorporating natural oils (pomegranate, Mahua) and conductive polymers (PANI) enhances bioactivity, antibacterial effects, and mechanical strength. Optimal formulations—such as 4% Sr/Zn-nHAp/collagen/PLGA, 50% HAp/PCL/gelatin, and SF/GelMA/nHA (7:2:1)—demonstrate improved osteogenic differentiation, apatite formation, cell viability, and in vivo bone regeneration, highlighting their promise for orthopedic applications. The performance comparison of electrospun HAP–polymer composite scaffolds is presented in Table 5.

**Table 5:** Performance comparison of electrospun HAP‑polymer composite scaffolds

S.No	Material system	Polymer type	HAP/Dopant	Young’s Modulus (GPa)	Tensile strength (MPa)	Cell viability (%)	Key bioactivity outcome	Primary limitation	References
1	nHAP/SF	Natural (Silk)	nHAP (50 mg)	0.15	–	Not reported	Supports BMSC development	Low modulus, not load-bearing	[81]
2	Ag–SrHAP/CS	Natural (Chitosan)	Ag⁺, Sr^2^⁺	–	–	Excellent (qualitative)	Osteoinductive + antibacterial	No quantitative mechanical data	[121]
3	PCL/GE/HAP 50%	Synthetic (PCL)	HAP (50% w/v)	–	–	> 85%	Increased bone formation in vivo (6 weeks)	High HAP content increases brittleness	[149]
4	Sr/Zn-nHAP/Col/PLGA	Synthetic (PLGA)	Sr^2^⁺, Zn^2^⁺ (4%)	9.91	–	–	Sustained ion release, apatite formation	Hardness (0.30 GPa) may limit ductility	[145]
5	PVA/tri-metal HAP	Synthetic (PVA)	Cu^2^⁺, Mn^2^⁺, Sr^2^⁺ (40%)	–	5.67	74% (at 10 μg/mL)	Antibacterial (S. aureus 20.8 mm, E. coli 21.8 mm)	Moderate cell viability, IC50 21.66 μg/mL	[153]
6	SF/GelMA/nHA 7:2:1	Natural/Synthetic blend	nHA (in situ)	–	–	Excellent	110% elongation, apatite formation in 3 days	Complex fabrication, multi-step process	[152]
7	COL/SF/CaO–SiO₂	Natural blend	Bioactive glass (20 ± 5 nm)	–	–	–	Enhanced vascularization + osteogenesis in osteoporotic rat model	No standalone HAP, complex ternary system	[133]
8	PHB/Nb₂O₅	Synthetic (PHB)	Nb₂O₅ (6%)	–	3 × increase over pure PHB	Non-toxic	Upregulated osteogenic genes, peak ALP activity	Nb₂O₅ is not HAP; different reinforcement mechanisms	[136]

The comparative analysis of electrospun HAP‑polymer composites reveals distinct performance trade‑offs. Natural polymer‑based scaffolds like nHAP/SF support bone marrow stem cell (BMSC) development but exhibit low modulus (0.15 GPa) and are not load‑bearing. Synthetic systems such as Sr/Zn‑nHAP/collagen/PLGA offer higher stiffness (Young’s modulus 9.91 GPa) with sustained ion release and apatite formation, though hardness (0.30 GPa) may limit ductility. PCL/GE/HAP (50% w/v) achieves > 85% cell viability and increased in vivo bone formation, but high HAP content raises brittleness. Antibacterial composites like PVA/tri‑metal HAP show tensile strength of 5.67 MPa and good inhibition zones against S. aureus and E. coli, yet exhibit moderate cell viability (74% at 10 μg/mL) with an IC50 of 21.66 μg/mL. Blended natural/synthetic scaffolds (SF/GelMA/nHA 7:2:1) provide excellent elongation (110%) and rapid apatite formation (3 days), but require complex multi‑step fabrication. Ternary natural blends (COL/SF/CaO–SiO₂) enhance vascularization and osteogenesis in osteoporotic rat models, though they lack standalone HAP. Finally, PHB/Nb₂O₅ composites increase tensile strength three‑fold and upregulate osteogenic genes, but Nb₂O₅ is not HAP and follows a different reinforcement mechanism. Overall, no single composition meets all clinical requirements; material selection depends on the targeted balance between bioactivity, mechanical robustness, and processability. Electrospun nanofibrous scaffolds offer large surface area, porosity, drug delivery. Mimic ECM, promote cell adhesion/proliferation/differentiation. Polymer‑nanoparticle composites enhance bone regeneration [139]. Electrospun carbon nanofibers serve as scaffolds for bone regeneration, requiring porous structures to enable osteocyte differentiation [140]. This electrospinning technique fabricates ultrafine fibers that mimic the natural collagen network, making them suitable for bone, nerve, vascular, and skin tissue engineering; functionalization further enhances their properties [141]. Such biomimetic scaffolds have also been applied in wound healing, bone repair, and diabetes treatment—for example, PCL/spider silk composites improve fibroblast viability and wound closure, collagen‑chitosan enhances biomineralization, and silk fibroin maintains normoglycemia for up to 1 year [142]. Recently, PCL/silk fibroin/Ag‑NP nanofibers prepared via electrospinning showed that the addition of silk fibroin and silver nanoparticles reduces fiber diameter while improving stiffness and hydrophilicity [143]. Importantly, electrospun PVA/PVP blend membranes have demonstrated enhanced biocompatibility, with improved adhesion and proliferation of NIH 3T3 fibroblasts [144]. The electrospun scaffold composed of strontium/zinc co‑doped nano‑hydroxyapatite, collagen, and PLGA exhibited a uniform nanofibrous architecture with interconnected pores of approximately 2.9 µm. It demonstrated a Young’s modulus of 9.91 GPa, sustained release of Ca^2^⁺, Sr^2^⁺, and Zn^2^⁺ ions, promoted apatite formation, and maintained minimal pH fluctuations during degradation, making it suitable for orthopedic applications [145]. Electrospun PCL/gelatin/TiO₂ nanofibers demonstrated that the addition of titanium dioxide nanoparticles improves hydrophilicity, mechanical strength, and thermal properties. However, a concentration of 1 w/v% TiO₂ induced cell toxicity due to reactive oxygen species release, whereas lower concentrations were non‑toxic. Thus, an optimal TiO₂ concentration is essential to effectively enhance bone regeneration [146]. Electrospun hydroxyethyl cellulose/polyvinyl alcohol (HEC/PVA) scaffolds were coated with nano‑hydroxyapatite (nHA) via an alternate soaking process. The uniform nHA coating increased tensile strength and wettability while reducing elongation at break. Furthermore, the coated scaffolds promoted proliferation and differentiation of human osteosarcoma cells, demonstrating potential for bone regeneration [147]. Electrospun polyurethane (PU) scaffolds blended with Mahua oil (MO) and propolis (PP) exhibited reduced fiber diameter, increased mechanical strength, and improved hydrophilicity due to strong polymer‑oil interactions. The PU/MO/PP scaffolds were non‑toxic, showed low hemolysis, and enhanced fibroblast adhesion and proliferation, making them valuable candidates for bone tissue repair [148]. Electrospun polycaprolactone/gelatin (PCL/GE) scaffolds were blended with 10%, 30%, or 50% hydroxyapatite (HAp). The 50% HAp formulation provided the highest mechanical strength, exceeding 85% cell viability, and increased expression of collagen and osteopontin. In vivo rat models confirmed enhanced bone formation within 6 weeks, highlighting its promise for bone tissue engineering [149]. Piezoelectric poly(vinylidene fluoride‑co‑tetrafluoroethylene) (PVDF–TrFE) nanofibers filled with hydroxyapatite (HAp) mimicked natural collagen fibril diameters. The composite exhibited enhanced piezoelectric properties, surface characteristics, mineralization, and cell proliferation. HAp provided biological cues that promoted osteogenic differentiation of human mesenchymal stem cells, evidenced by increased ALP activity, calcium deposition, and osteogenic gene expression [150]. A double‑layered biomimetic nanofibrous scaffold encapsulating TGF‑β3 and functionalized with mussel adhesive protein (MAP) coating was developed. The double‑layer structure mimicked tendon and bone extracellular matrices, promoting multi‑tissue ingrowth. MAP coating enhanced biocompatibility and cell adhesion, while sustained TGF‑β3 release recruited stem cells and promoted chondrogenic differentiation via the PI3K‑Akt signaling pathway. In a rat rotator cuff tear model, the scaffold significantly improved cartilage regeneration, enthesis structure, and biomechanical properties [151]. Electrospun and photocrosslinked nanofibrous membranes composed of silk fibroin (SF), methacrylated gelatin (GelMA), and nano‑hydroxyapatite (nHA) at an optimal mass ratio of 7:2:1 exhibited 110% elongation at break, maintained nanofibrous structure for 28 days, and formed bone‑like apatite within 3 days. The membrane showed excellent biocompatibility, enhanced osteogenic differentiation of human bone marrow mesenchymal stem cells (hBMSCs) in vitro, and improved bone regeneration in rat critical‑sized calvarial defects in vivo, making it a promising candidate for guided bone regeneration [152]. Electrospun polyvinyl alcohol (PVA) nanofibrous mats incorporated tri‑metal (Cu^2^⁺, Mn^2^⁺, Sr^2^⁺) co‑doped hydroxyapatite (HAP) at varying ratios, with the optimal composite being 40% doped HAP in PVA (TPE‑46). The scaffolds achieved approximately 99% porosity within 48 h, exhibited excellent blood compatibility (< 5% hemolysis), hydrophilic properties, and an ultimate tensile strength of 5.67 MPa. They demonstrated superior antibacterial activity against S. aureus (20.8 mm) and E. coli (21.8 mm), with 74% cell viability at 10 μg/mL and an IC50 of 21.66 μg/mL [153]. Similar to Ref 153, the electrospun and photocrosslinked SF/GelMA/nHA (7:2:1) membrane achieved 110% elongation at break, preserved nanofibrous structure for 28 days, and formed bone‑like apatite within 3 days. It exhibited excellent biocompatibility, enhanced hBMSC osteogenic differentiation, and improved rat calvarial bone regeneration, confirming its suitability for guided bone regeneration [152]. A photocrosslinkable natural hydrogel nanofibrous membrane (SFMA‑Lap@AcBP) was fabricated by combining electrospinning with hydrogel technology using methacrylated silk fibroin, a photoinitiator, and acrylate bisphosphonate. This membrane mimics periosteum structure and function. AcBP is covalently attached and released upon degradation to promote bone regeneration. The membrane traps Mg^2^⁺ and Ca^2^⁺ ions in vivo, providing a synergistic effect. It offers excellent cytocompatibility and ECM‑like cues for cell adhesion, proliferation, and differentiation without requiring exogenous cells or growth factors. In rat cranial defect models, it significantly enhanced new bone formation, acting as an efficient bionic periosteum [154]. Recently 3D bioprinted and electrospun polyurethane scaffolds were reviewed for bone tissue engineering. Polyurethane offers superior mechanical properties compared to other biomaterials. Electrospinning and bioprinting provide excellent control over scaffold geometry and porosity, enabling tunable cell viability and growth. Advantages include creating optimal microenvironments for cell adhesion/proliferation, offering personalized scaffolds based on defect size/location, and incorporating osteoinductive/osteogenic agents. Identified knowledge gaps include challenges with cell‑loaded polyurethane bioinks (non‑hydrogel structure) and the lack of 3D cell culture in electrospun scaffolds [155]. Electrospun poly(ε‑caprolactone) (PCL) nanofiber scaffolds incorporating 15% w/w 45S5 bioactive glass (BG) particles and pomegranate seed oil (PSO) at 5, 10, and 15% w/w relative to PCL were evaluated. The nanofibers with 15% PSO and 15% BG showed the highest formation of a hydroxyapatite‑like layer after 28 days in simulated body fluid compared to lower PSO concentrations. MTT assays using L929 fibroblast cells confirmed cytocompatibility. The combination of BG and PSO in PCL nanofibers demonstrates potential for improving bone tissue regeneration strategies [156]. A comprehensive summary of these electrospun nanofibrous scaffolds, including materials, composites, and key findings, is provided in Table 6.

**Table 6:** Summary of electrospun nanofibrous scaffolds for bone tissue engineering: key materials/composites and key findings/focus

S.NO	Materials/Composite	Key findings/Focus	References
1	Polymeric nanofibers, Carbon nanofibers, Mineralized nanofibers (mimicking bone ECM: collagen fibers + hydroxyapatite crystals)	Highlights that bioceramics combined with mineralized polymeric and carbon nanofibers prepared via mineralization techniques are promising scaffold.Their key advantages include: (1) high similarity to the bone extracellular matrix (ECM), (2) high surface-to-volume ratio for better cell interactions, and (3) the ability to precisely tailor physicochemical features. This paper specifically reviews the fabrication methods and applications of these mineralized nanofibers to augment bone healing	[138]
2	Natural polymeric nanofibers, synthetic polymeric nanofibers, organic/inorganic nanoparticle composites (incorporated into electrospun nanofibrous scaffolds)	Electrospun nanofibrous scaffolds are highly efficient for bone regeneration due to their large surface area, high porosity, and drug loading capacity. Key findings include: (1) they mimic the natural extracellular matrix (ECM), promoting cell adhesion, proliferation, and differentiation. (2) They serve as effective carriers for drug/growth factor delivery. (3) Composite scaffolds combining organic polymers with inorganic nanoparticles significantly enhance bone tissue regeneration. The review also discusses future prospects and applicability to other tissue regeneration strategies	[139]
3	Electrospun nanofibers (with a specific focus on carbon nanofibers produced by the electrospinning method)	Introduces the potential use of electrospun nanofibers as scaffolds for bone regenerative medicine. Key points include: (1) Scaffolds require porous materials that allow cell differentiation into osteocytes; (2) Electrospinning is an effective method for producing nano-sized fibers by applying high voltage to a polymer solution; (3) Carbon nanofibers produced via electrospinning are highlighted as a growing topic of interest for bone-regeneration therapy	[140]
4	Electrospun nanofibers (fabricated from polymer media), functionalized with various materials, and assembled into 3D structures or combined with other nanomaterials	Reporting the recent progress in electrospun nanofibers for biomedical applications. Key points include: (1) electrospinning is a versatile technique to fabricate ultrafine fibers that mimic the collagen fiber network of the natural extracellular matrix; (2) These nanofibers are suitable for bone cell proliferation, nerve regeneration, vascular tissue engineering, and skin tissue engineering; (3) Functionalization with different materials enhances their properties; (4) The review also highlights challenges and future outlooks for improving electrospun nanofiber applications	[141]
5	Collagen–carbohydrate hybrids with graphene; electrospun nanofibers (PCL, spider silk, polymeric carbohydrates); collagen–chitosan; silk fibroin + pancreatic β-cells; fibrin scaffolds; CMGG hydrogels; hydroxyapatite–polymer composites; decellularized ECM	Biomimetic scaffolds for wound healing, bone repair, and diabetes. Key results: (1) electrospun scaffolds improve fibroblast viability by 90% and wound closure by 25% (PCL/spider silk, 21 days). (2) Collagen–chitosan enhances biomineralization and mechanical stability by 25% over collagen–alginate. (3) β-cell seeded silk fibroin maintains normoglycemia for 1 year; fibrin scaffolds enable 12-month insulin independence. (4) CMGG reduces burn healing time by ~ 95%. Hydroxyapatite composites improve osteoconductivity. Future focus: vascularization, clinical translation, smart biomaterials	[142]
6	Polycaprolactone (PCL) Silk fibroin (SF) extracted from *Bombyx mori* cocoons Silver nanoparticles (Ag-NPs) PCL15/fibroin/Ag-NPs nanofibers (fabricated via electrospinning)	Addition of SF and Ag-NPs reduced fiber diameter, improved stiffness, and enhanced hydrophilicity Strong intermolecular interactions among PCL, fibroin, and Ag-NPs confirmed by FTIR	[143]
7	Aqueous solutions of polyvinyl alcohol (PVA) and polyvinylpyrrolidone (PVP), along with their physical blends, were electrospun using the gravity method. The resulting nanofibrous membranes exhibited complete solvent evaporation and uniform blend formation	The membranes showed bead-free morphology and enhanced biocompatibility, as confirmed by improved adhesion and proliferation of mouse NIH 3T3 fibroblasts. This positions the material as a promising scaffold for tissue engineering applications	[144]
8	The scaffold is a biomimetic electrospun composite made of strontium/zinc co-doped nano–hydroxyapatite (Sr/Zn-nHAp), collagen, and poly(lactic–co-glycolic acid) (PLGA) polymer. The specific formulation of 4% Sr/Zn-doped nHAp with collagen and PLGA was fabricated using electrospinning for bone tissue engineering (BTE) applications	The 4% Sr/Zn-nHAp–collagen–PLGA scaffold exhibited a uniform nanofibrous structure with interconnected pores (2.9 µm) and mechanical properties (Young’s modulus 9.91 GPa, hardness 0.30 GPa) comparable to cancellous bone. It showed substantial bioactivity with apatite formation, sustained release of Ca^2^⁺, Sr^2^⁺, and Zn^2^⁺ ions, and a controlled degradation profile with minimal pH fluctuations, making it a promising candidate for orthopedic applications	[145]
9	Electrospun PCL/gelatin/TiO₂ nanofibrous scaffolds with three TiO₂ nanoparticle concentrations (0.06, 0.6, and 1 w/v%)	TiO₂ addition improved hydrophilicity, mechanical, and thermal properties. However, 1 w/v% TiO₂ caused cell toxicity due to reactive oxygen release, while lower concentrations showed no toxicity. An optimum TiO₂ concentration enhances bone regeneration	[146]
10	Electrospun hydroxyethyl cellulose/polyvinyl alcohol (HEC/PVA) nanofibrous scaffolds coated with nanohydroxyapatite (nHA) via an alternate soaking process	nHA coating formed uniformly over nanofibers, enhancing tensile strength and wettability while reducing elongation at break. The coated scaffolds promoted proliferation and differentiation of human osteosarcoma cells, making them a potential candidate for bone regeneration	[147]
11	lectrospun polyurethane (PU) scaffolds blended with Mahua oil (MO) and propolis (PP)	Adding MO and PP reduced fiber diameter, enhanced mechanical strength, and showed strong polymer–oil interactions. The PU/MO/PP scaffold exhibited hydrophilic behavior, non-toxicity, low hemoglobin release, and improved blood clotting time. Enhanced fibroblast cell adhesion and proliferation make it a valuable candidate for bone tissue repair	[148]
12	Electrospun polycaprolactone (PCL)/gelatin (GE) scaffolds blended with three concentrations of hydroxyapatite (HAp): 10%, 30%, and 50% (w/v)	The 50% HAp–PCL/GE blend possessed the highest mechanical strength. It increased cell viability (> 85%), proliferation, cell-surface attachment, and higher expression of collagen and osteopontin. In vivo rat models confirmed increased bone formation within 6 weeks, making it highly promising for bone tissue engineering	[149]
13	Electrospun piezoelectric poly(vinylidene fluoride–co-tetrafluoroethylene) (PVDF–TrFE) nanofibers filled with hydroxyapatite (HAp)	The PVDF–TrFE/HAp nanofibers mimicked collagen fibril diameters and exhibited enhanced piezoelectric properties and surface characteristics. This improved mineralization and cell proliferation. HAp provided biological cues that enhanced osteogenic differentiation of human mesenchymal stem/stromal cells (MSCs), as evidenced by increased ALP activity, calcium deposition, and osteogenic gene expression, demonstrating potential for bone tissue regeneration	[150]
14	Double-layered biomimetic nanofibrous scaffold encapsulating TGF-β3, functionalized with a mussel adhesive protein (MAP) coating	The double-layer structure mimicked tendon and bone extracellular matrices, promoting multiple tissue ingrowth. MAP coating enhanced biocompatibility and cell adhesion. Sustained TGF-β3 release promoted stem cell recruitment and chondrogenic differentiation via the PI3K–Akt signaling pathway. In a rat rotator cuff tear model, the scaffold significantly enhanced cartilage regeneration, restoring enthesis structure and biomechanical properties	[151]
15	Electrospun and photocrosslinked nanofibrous membranes composed of silk fibroin (SF), anhydride-modified gelatin (GelMA), and nano–hydroxyapatite (nHA) synthesized in situ. The optimal mass ratio was 7:2:1 (SF:GelMA:nHA)	The 7:2:1 membrane exhibited 110% elongation at break, maintained nanofibrous structure for 28 days, and formed bone-like apatite within 3 days. It showed excellent biocompatibility and enhanced osteogenic differentiation of hBMSCs in vitro, and improved bone regeneration in rat critical-sized calvarial defects in vivo, making it a promising candidate for guided bone regeneration	[152]
16	Electrospun polyvinyl alcohol (PVA) nanofibrous mats incorporating tri-metal (Cu^2^⁺, Mn^2^⁺, Sr^2^⁺) co-doped hydroxyapatite (HAP) at varying weight ratios (2:8, 4:6, and 5:5). The optimal composite was 40% tri-metal HAP in PVA (TPE-46)	The scaffolds achieved ~ 99% porosity within 48 h, excellent blood compatibility (< 5% hemolysis), and hydrophilic properties. The TPE-46 composite (40% doped HAP) showed ultimate tensile strength of 5.67 MPa and superior antibacterial activity against S. aureus (20.8 mm) and E. coli (21.8 mm). It exhibited 74% cell viability at 10 μg/mL with an IC50 of 21.66 μg/mL, demonstrating potential for bone tissue engineering	[153]
17	Electrospun and photocrosslinked nanofibrous membranes of silk fibroin (SF), anhydride-modified gelatin (GelMA), and nano-hydroxyapatite (nHA) synthesized in situ. The optimal mass ratio was SF:GelMA:nHA = 7:2:1	The 7:2:1 membrane achieved 110% elongation at break, preserved nanofibrous structure for 28 days, and formed bone-like apatite within 3 days. It exhibited excellent biocompatibility and enhanced osteogenic differentiation of hBMSCs in vitro, and improved bone regeneration in rat critical-sized calvarial defects in vivo, making it a promising candidate for guided bone regeneration	[152]
18	A photocrosslinkable natural hydrogel nanofibrous membrane (SFMA-Lap@AcBP) based on methacrylated silk fibroin (SFMA), photoinitiator (Lap), and acrylate bisphosphonate (AcBP), fabricated by combining electrospinning with hydrogel technology	The membrane mimics periosteum structure and function. AcBP is covalently attached and released upon degradation to promote bone regeneration. The membrane traps Mg^2^⁺ and Ca^2^⁺ ions in vivo, providing a synergistic effect. It offers excellent cytocompatibility and extracellular matrix-like cues for cell adhesion, proliferation, and differentiation without needing exogenous cells or growth factors. In rat cranial defect models, it significantly enhanced new bone formation, making it an efficient bionic periosteum for bone regeneration	[154]
19	3D bioprinted and electrospun polyurethane scaffolds for bone tissue engineering	Polyurethane offers superior mechanical properties compared to other biomaterials. Electrospinning and bioprinting provide excellent control over scaffold geometry and porosity, enabling tunable cell viability and growth. Advantages include creating optimal microenvironments for cell adhesion/proliferation, offering personalized scaffolds based on defect size/location, and incorporating osteoinductive/osteogenic agents. Knowledge gaps include challenges with cell-loaded polyurethane bioinks (non-hydrogel structure) and lack of 3D cell culture in electrospun scaffolds	[155]
20	Electrospun poly(ε-caprolactone) (PCL) nanofiber scaffolds incorporating 15% w/w 45S5 bioactive glass (BG) particles and pomegranate seed oil (PSO) at varying concentrations (5, 10, and 15% w/w relative to PCL)	The nanofiber with 15% PSO and 15% BG showed the highest formation of hydroxyapatite-like layer after 28 days in simulated body fluid compared to lower PSO concentrations. MTT assay using L929 fibroblast cells confirmed cytocompatibility. The combination of BG and PSO in PCL nanofibers demonstrates potential for improving bone tissue regeneration strategies	[156]

From the table, we see the electrospun nanofibers mimic bone ECM, offering high surface area, porosity, and drug delivery. Mineralized polymeric/carbon nanofibers enhance bone healing. Natural/synthetic polymer composites with HAp, bioactive glass, or doped HAp (Sr^2^⁺, Zn^2^⁺, Cu^2^⁺) improve mechanical strength, bioactivity, and osteogenic differentiation. Optimal formulations (e.g., 50% HAp/PCL/gelatin, 4% Sr/Zn-nHAp/collagen/PLGA, SF/GelMA/nHA 7:2:1) show > 85% cell viability, apatite formation, and in vivo bone regeneration. Additives such as TiO₂, Ag-NPs, or natural oils provide antibacterial effects but require careful dosing to avoid toxicity. Advanced designs (double-layer, piezoelectric, 3D bioprinted) enable tendon-bone healing and personalized scaffolds.

## Conclusions and future perspectives

The objective of this research was to survey recent advances in the field of bone tissue engineering (BTE) using biomimetic nanostructured polymeric composites. Numerous synthetic and biopolymers, such as PGA, PLGA, PLA, PLLA, silk fibroin, gelatin, chitosan, and cellulose, have been considered as viable choices for BTE applications because of their biodegradability and ease of fabrication using the electrospinning (E-spin) technique. However, the biomedical application of biomimetic nanofibrous composites requires the successful interaction of cells, biological signals, and biomaterials. Nevertheless, there are still many unresolved issues and uncharted areas that can significantly impact the function of nanostructured materials in bone regeneration. The development of effective regeneration technologies necessitates a basic grasp of both materials and biological sciences. Better bio-mimicking of the natural process of bone tissue regeneration—such as the linkage between angiogenesis and osteogenesis, which may require the recruitment and differentiation of progenitor cells—should be the focus of future studies. Even if it is challenging to replicate nature, new scientific and technological discoveries indicate that bone composites supporting both local and systemic biological processes may be possible.

In order to construct bone composites in the future, it will be crucial to choose the right composite materials, consider their shape, pore size and distribution, and the release rate of biomolecules. The last 10 years have seen the emergence of electrospun composites as viable options for creating materials that closely match the natural extracellular matrix (ECM) and can be effectively implanted for BTE. Nevertheless, there is still much to learn about the combination of these elements in the form of nanocomposites. To create composite materials that are both stronger and more resilient, a hierarchical design spanning multiple length scales must be incorporated, including micro-level structures to influence crack paths and create toughness, as well as the nano-level to generate strength (i.e., to mimic composite deformation of collagen and HAp). The limitations of processing techniques are one factor that has hindered the development of innovative composites to produce structures with a multidimensional design. Combining these technologies with nanomaterials remains a challenge. An ideal scaffold could ultimately incorporate a number of materials and methods. Biodegradable and sustainable polymers, including cellulose and its derivatives, are highly sought after and may open up new possibilities for bone tissue engineering. With the development of new production techniques, such as electrospinning and electrospraying, it may be possible to bio-mimic the natural bone extracellular matrix (ECM) using various polymeric, nano-apatite, and calcium phosphate materials. Ultimately, it has been shown that in order to achieve the composite properties needed for BTE applications, it is crucial to combine a range of biomaterials, including polymeric and bioceramic materials, as well as other bioactive materials.

## Data Availability

The datasets used and/or analyzed during the current study available from the corresponding author on reasonable request.
